# Abortion Surveillance — United States, 2020

**DOI:** 10.15585/mmwr.ss7110a1

**Published:** 2022-11-25

**Authors:** Katherine Kortsmit, Antoinette T. Nguyen, Michele G. Mandel, Elizabeth Clark, Lisa M. Hollier, Jessica Rodenhizer, Maura K. Whiteman

**Affiliations:** 1 Division of Reproductive Health, National Center for Chronic Disease Prevention and Health Promotion, CDC

## Abstract

**Problem/Condition:**

CDC conducts abortion surveillance to document the number and characteristics of women obtaining legal induced abortions and number of abortion-related deaths in the United States.

**Period Covered:**

2020.

**Description of System:**

Each year, CDC requests abortion data from the central health agencies for the 50 states, the District of Columbia, and New York City. For 2020, a total of 49 reporting areas voluntarily provided aggregate abortion data to CDC. Of these, 48 reporting areas provided data each year during 2011–2020. Census and natality data were used to calculate abortion rates (number of abortions per 1,000 women aged 15–44 years) and ratios (number of abortions per 1,000 live births), respectively. Abortion-related deaths from 2019 were assessed as part of CDC’s Pregnancy Mortality Surveillance System (PMSS).

**Results:**

A total of 620,327 abortions for 2020 were reported to CDC from 49 reporting areas. Among 48 reporting areas with data each year during 2011–2020, in 2020, a total of 615,911 abortions were reported, the abortion rate was 11.2 abortions per 1,000 women aged 15–44 years, and the abortion ratio was 198 abortions per 1,000 live births. From 2019 to 2020, the total number of abortions decreased 2% (from 625,346 total abortions), the abortion rate decreased 2% (from 11.4 abortions per 1,000 women aged 15–44 years), and the abortion ratio increased 2% (from 195 abortions per 1,000 live births). From 2011 to 2020, the total number of reported abortions decreased 15% (from 727,554), the abortion rate decreased 18% (from 13.7 abortions per 1,000 women aged 15–44 years), and the abortion ratio decreased 9% (from 217 abortions per 1,000 live births).

In 2020, women in their 20s accounted for more than half of abortions (57.2%). Women aged 20–24 and 25–29 years accounted for the highest percentages of abortions (27.9% and 29.3%, respectively) and had the highest abortion rates (19.2 and 19.0 abortions per 1,000 women aged 20–24 and 25–29 years, respectively). By contrast, adolescents aged <15 years and women aged ≥40 years accounted for the lowest percentages of abortions (0.2% and 3.7%, respectively) and had the lowest abortion rates (0.4 and 2.6 abortions per 1,000 women aged <15 and ≥40 years, respectively). However, abortion ratios were highest among adolescents (aged ≤19 years) and lowest among women aged 25–39 years.

Abortion rates decreased from 2011 to 2020 among all age groups. The decrease in abortion rate was highest among adolescents compared with any other age group. From 2019 to 2020, abortion rates decreased or did not change for all age groups. Abortion ratios decreased from 2011 to 2020 for all age groups, except adolescents aged 15–19 years and women aged 25–29 years for whom abortion ratios increased. The decrease in abortion ratio was highest among women aged ≥40 years compared with any other age group. From 2019 to 2020, abortion ratios decreased for adolescents aged <15 years and women aged ≥35 and increased for women 15–34 years.

In 2020, 80.9% of abortions were performed at ≤9 weeks’ gestation, and nearly all (93.1%) were performed at ≤13 weeks’ gestation. During 2011–2020, the percentage of abortions performed at >13 weeks’ gestation remained consistently low (≤9.2%). In 2020, the highest percentage of abortions were performed by early medical abortion at ≤9 weeks’ gestation (51.0%), followed by surgical abortion at ≤13 weeks’ gestation (40.0%), surgical abortion at >13 weeks’ gestation (6.7%), and medical abortion at >9 weeks’ gestation (2.4%); all other methods were uncommon (<0.1%). Among those that were eligible (≤9 weeks’ gestation), 63.9% of abortions were early medical abortions. In 2019, the most recent year for which PMSS data were reviewed for pregnancy-related deaths, four women died as a result of complications from legal induced abortion.

**Interpretation:**

Among the 48 areas that reported data continuously during 2011–2020, overall decreases were observed during 2011–2020 in the total number, rate, and ratio of reported abortions. From 2019 to 2020, decreases also were observed in the total number and rate of reported abortions; however, a 2% increase was observed in the total abortion ratio.

**Public Health Action:**

Abortion surveillance can be used to help evaluate programs aimed at promoting equitable access to patient-centered quality contraceptive services in the United States to reduce unintended pregnancies.

## Introduction

This report summarizes data on legal induced abortions for 2020 that were provided voluntarily to CDC by the central health agencies of 49 reporting areas (47 states, the District of Columbia, and New York City, excluding California, Maryland, and New Hampshire) and comparisons over time for the 48 reporting areas that reported each year during 2011–2020 (47 states and New York City). This report also summarizes abortion-related deaths reported voluntarily to CDC for 2019 as part of the Pregnancy Mortality Surveillance System (PMSS). Since 1969, CDC has conducted abortion surveillance to document the number and characteristics of women obtaining legal induced abortions in the United States. After nationwide legalization of abortion in 1973, the total number, rate (number of abortions per 1,000 women aged 15–44 years), and ratio (number of abortions per 1,000 live births) of reported abortions increased rapidly, reaching the highest levels in the 1980s, before decreasing at a slow yet steady pace (*1*,*2*). During 2006–2008, a break occurred in the previously sustained pattern of decrease (*3*,*4*), although this break was followed in subsequent years by even greater decreases (*5*,*6*). In 2017, the total number, rate, and ratio of reported abortions reached historic lows (*5*); however, from 2018 to 2019, 1%–3% increases were observed across all measures (*7*). Nonetheless, despite the overall decreases, abortion incidence and practices have varied over the years and continue to vary across subpopulations (*8*–*12*), highlighting the utility of continued surveillance.

## Methods

### Description of the Surveillance System

Each year, CDC requests aggregate data from the central health agencies of the 50 states, the District of Columbia, and New York City to document the number and characteristics of women obtaining legal induced abortions in the United States. Not all persons who obtain abortions identify as women; the term “women” has been maintained in this report to be consistent with the collection and reporting of denominator data used to calculate abortion rates and ratios. This report contains data voluntarily reported to CDC as of August 19, 2022. For the purpose of surveillance, legal induced abortion is defined as an intervention performed within the limits of state law by a licensed clinician (e.g., a physician, nurse-midwife, nurse practitioner, or physician assistant) intended to terminate a suspected or known intrauterine pregnancy and that does not result in a live birth. This definition excludes management of intrauterine fetal death, early pregnancy failure/loss, ectopic pregnancy, or retained products of conception. All abortions in this report are considered to be legally induced unless stated otherwise.

In most states and jurisdictions, collection of abortion data are facilitated by a legal requirement for hospitals, facilities, or physicians to report abortions to a central health agency (*13*); however, reporting is not complete in all areas, including in certain areas with reporting requirements (*14*). Because the reporting of abortion data to CDC is voluntary, many reporting areas have developed their own data collection forms and might not collect or provide all the information requested by CDC. As a result, the level of detail reported by CDC might vary from year to year and by reporting area. To encourage uniform collection of data, CDC has collaborated with the National Association for Public Health Statistics and Information Systems (NAPHSIS) to develop reporting standards and provide technical guidance for vital statistics personnel who collect and summarize abortion data within the United States.

### Variables and Categorization of Data

Each year, CDC sends a suggested template to central health agencies in the United States for compilation of aggregate abortion data among women obtaining legal induced abortions. Aggregate abortion numbers, without individual-level records, are requested for the following variables:

- Age group in years of women obtaining legal induced abortions (<15, 15–19 [age group and by individual year], 20–24, 25–29, 30–34, 35–39, or ≥40)
- Gestational age of pregnancy in completed weeks at the time of abortion (≤6, 7–20 by individual week, or ≥21)
- Race (Black, White, or other [including Asian, Pacific Islander, other races, and multiple races]), ethnicity (Hispanic or non-Hispanic), and race by ethnicity
- Method type (surgical abortion, intrauterine instillation, medical [nonsurgical] abortion, or hysterectomy/hysterotomy)
- Marital status (married [including currently married or separated] or unmarried [including never married, widowed, or divorced])
- Number of previous live births (zero, one, two, three, or four or more)
- Number of previous induced abortions (zero, one, two, or three or more)
- Residence (the state, jurisdiction, territory, or foreign country in which the women obtaining the abortion lived, or, if additional details are unavailable, in-reporting area versus out-of-reporting area)

In addition, the template provided by CDC requests that aggregate abortion numbers for certain variables be cross-tabulated by a second variable. The cross-tabulations presented in this report include weeks of gestation separately by method type, by age group, and by race or ethnicity.

Beginning with 2014 data, instead of reporting the clinician’s estimates of gestational age or estimates of gestational age based on last menstrual period, certain areas have reported “probable postfertilization age,” “clinician’s estimate of gestation based on date of conception,” and “probable gestational age” to CDC. To ensure consistency between data reported as postfertilization age and the data collection practices for gestational age recommended by CDC’s National Center for Health Statistics (*15*), 2 weeks were added to probable postfertilization age. This method was used to account for time after last menstrual period until ovulation in a standard 28-day cycle because fertilization occurs around the time of ovulation (*16*). No modifications were made to data reported as clinician’s estimate of gestational age based on date of conception or data reported as probable gestational age.

In this report, medical and surgical abortions are further categorized by gestational age when available in the categories reported to CDC. Early medical abortion is defined as the administration of medications (typically mifepristone followed by misoprostol) to induce an abortion at ≤9 completed weeks’ gestation consistent with U.S. Food and Drug Administration (FDA) labeling for mifepristone that was implemented in 2016 (*17*). CDC collects information only on the estimated number of weeks (not days) of gestation and acknowledges the conventional use of completed weeks of gestation to describe pregnancy duration; therefore, CDC’s category of ≤9 weeks’ gestation includes abortions through 9 weeks and 6 days. Medications (typically serial prostaglandins, sometimes administered after mifepristone) also might be used to induce an abortion at >9 weeks’ gestation. Surgical abortions, which include uterine aspiration (i.e., dilation and curettage, aspiration curettage, suction curettage, manual vacuum aspiration, menstrual extraction, or sharp curettage) and dilation and evacuation procedures, are categorized as having been performed at ≤13 weeks’ gestation or at >13 weeks’ gestation because of differences in surgical technique at these gestational ages (*18*). Finally, because intrauterine instillations are unlikely to be performed early in gestation (*19*), abortions reported to have been performed by intrauterine instillation at ≤12 weeks’ gestation are excluded from calculation of the percentage of abortions by known method type and are grouped with unknown type.

### Measures of Abortion

Four measures of abortion are presented in this report: 1) the number of abortions in a given population, 2) the percentage of abortions by selected characteristics, 3) the abortion rate (number of abortions per 1,000 women within a given population), and 4) the abortion ratio (number of abortions per 1,000 live births within a given population). Abortion rates adjust for differences in population size. Abortion ratios measure the relative number of pregnancies in a population that end in abortion compared with live birth.

The U.S. Census Bureau estimates of the resident female population were used as the denominator for calculating abortion rates (*20*–*29*). Overall abortion rates were calculated from the population of women aged 15–44 years living in the reporting areas that provided continuously reported data. For adolescents aged <15 years, abortion rates were calculated using the number of adolescents aged 13–14 years as the denominator; for women aged ≥40 years, abortion rates were calculated using the number of women aged 40–44 years as the denominator. For the calculation of abortion ratios, live birth data were obtained from CDC natality files and included births to women of all ages living in the reporting areas that provided abortion data (*30*,*31*). For calculation of the total abortion rates and total ratios only, women with unknown data on selected characteristics (e.g., age, race or ethnicity, and marital status) were distributed according to the distribution of abortions among those with known information on the characteristic. For calculation of totals only, abortions for women with an unknown gestational age of pregnancy but known method type were distributed according to the distribution of abortions among those with known information on method type by gestational age to the following categories: surgical, ≤13 weeks’ gestation; surgical, >13 weeks’ gestation; medical, ≤9 weeks’ gestation; and medical, >9 weeks’ gestation.

### Data Presentation and Analysis

This report provides aggregate and reporting area–specific abortion numbers, rates, and ratios for the 49 areas that reported to CDC for 2020, which excluded California, Maryland, and New Hampshire. In addition, this report describes characteristics of women who obtained abortions in 2020. The data in this report are presented by the reporting area in which the abortions were performed.

The completeness and quality of data received vary by year, by variable, and by reporting area; this report only describes the characteristics of women obtaining abortions in reporting areas that met CDC reporting standards (i.e., reported at least 20 abortions overall, provided data categorized in accordance with requested variables, and had <15% unknown values for a given characteristic). Cells with a numerical value in the range of 1–4 and cells that would allow for calculation of these values have been suppressed in this report to maintain confidentiality in tables presented by reporting area of occurrence.

The percentage change in abortion measures (number, rate, and ratio of reported abortions) from the most recent past year (2019 to 2020) and during the 10-year period of analysis (2011–2020) were calculated for the 48 areas that reported every year during 2011–2020. The percentage change was also calculated and reported for abortions by age group, weeks of gestation, and early medical abortions (≤9 completed weeks’ gestation) for areas that met reporting standards for these variables every year during 2011–2020. As a result, aggregate measures for 2020 in percentage change analyses might differ from the point estimates reported for 2020. These data describe the percentage changes in abortion measures over time and abortions measures among groups for each characteristic. No statistical testing was performed. Comparisons do not imply statistical significance, and lack of comment regarding the difference between values does not imply that no statistically significant difference exists.

### Abortion Mortality

CDC has reported data on abortion-related deaths periodically since information on abortion mortality first was included in the 1972 abortion surveillance report (*7*,*32*). An abortion-related death is defined as a death resulting from a direct complication of an abortion (legal or illegal), an indirect complication caused by a chain of events initiated by an abortion, or an aggravation of a pre-existing condition by the physiologic effects of abortion. An abortion is categorized as legal when it is performed by a licensed clinician within the limits of state law.

Since 1987, CDC has monitored abortion-related deaths through PMSS, which includes data from all 50 states, the District of Columbia, and New York City (*33*). Sources of data to identify abortion-related deaths have included state vital records; media reports, including computerized searches of full-text newspaper and other print media databases; and individual case reports by public health agencies, including maternal mortality review committees, health care providers and provider organizations, private citizens, and citizen groups. For each death that is possibly related to abortion, CDC requests clinical records and autopsy reports. Two medical epidemiologists independently review these reports to determine the cause of death and whether the death was abortion related. Discrepancies are discussed and resolved by consensus. Each death is categorized by abortion type as legal induced, illegal induced, spontaneous, or unknown type.

This report provides PMSS data on induced abortion-related deaths that occurred in 2019, the most recent year for which PMSS data are available. For 1998–2019, abortion surveillance data reported to CDC cannot be used alone to calculate national case-fatality rates for legal induced abortions (number of legal induced abortion-related deaths per 100,000 reported legal induced abortions in the United States) because eight reporting areas did not report abortion data every year during this period (Alaska, 1998–2000; California, 1998–2019; the District of Columbia, 2016; Louisiana, 2005; Maryland, 2007–2019; New Hampshire, 1998–2019; Oklahoma, 1998–1999; and West Virginia, 2003–2004). Thus, denominator data for calculation of national legal induced abortion case-fatality rates were obtained from a published report by the Guttmacher Institute that includes estimated total numbers of abortions in the United States from a national survey of abortion-providing facilities (*6*,*34*). The case-fatality rate was calculated using denominator data for 2019. Because rates determined on the basis of a numerator <20 are unstable (*35*), national case-fatality rates for legal induced abortion were calculated for consecutive 5-year periods during 1973–2012 and then for a consecutive 7-year period during 2013–2019.

## Results

### Total Abortions Reported to CDC by Occurrence

Among the 49 reporting areas that provided data for 2020, a total of 620,327 abortions were reported. Of these abortions, 615,911 (99.3%) were from 48 reporting areas that provided data every year during 2011–2020. In 2020, these continuously reporting areas had an abortion rate of 11.2 abortions per 1,000 women aged 15–44 years and an abortion ratio of 198 abortions per 1,000 live births (Table 1). From 2019 to 2020, the total number of reported abortions decreased 2% (from 625,346 total abortions), the abortion rate decreased 2% (from 11.4 abortions per 1,000 women aged 15–44 years), and the abortion ratio increased 2% (from 195 abortions per 1,000 live births). From 2011 to 2020, the total number of reported abortions decreased 15% (from 727,554), the abortion rate decreased 18% (from 13.7 abortions per 1,000 women aged 15–44 years), and the abortion ratio decreased 9% (from 217 abortions per 1,000 live births) (Figure).

**TABLE 1:** Number, percentage, rate,* and ratio^†^ of reported abortions — selected reporting areas, United States, 2011–2020

Year	Selected reporting areas^§^	Continuously reporting areas^¶^		
	No.	No. (%)**	Rate	Ratio
**2011**	730,322	727,554 (99.6)	13.7	217
**2012**	699,202	696,587 (99.6)	13.1	208
**2013**	664,435	661,874 (99.6)	12.4	198
**2014**	652,639	649,849 (99.6)	12.1	192
**2015**	638,169	636,902 (99.8)	11.8	188
**2016**	623,471	623,471 (100.0)	11.6	186
**2017**	612,719	609,095 (99.4)	11.2	185
**2018**	619,591	614,820 (99.2)	11.3	189
**2019**	629,898	625,346 (99.3)	11.4	195
**2020**	620,327	615,911 (99.3)	11.2	198

* Number of abortions per 1,000 women aged 15–44 years.

^†^ Number of abortions per 1,000 live births.

^§^ For each given year, excludes reporting areas that did not report that year’s abortion numbers to CDC: California (2011–2020), the District of Columbia (2016), Maryland (2011–2020), and New Hampshire (2011–2020).

^¶^ For all years, excludes reporting areas that did not report abortion numbers every year during the analysis period: California, the District of Columbia, Maryland, and New Hampshire.

** Abortions from areas that reported every year during the analysis period as a percentage of all reported abortions for a given year.

**FIGURE:**
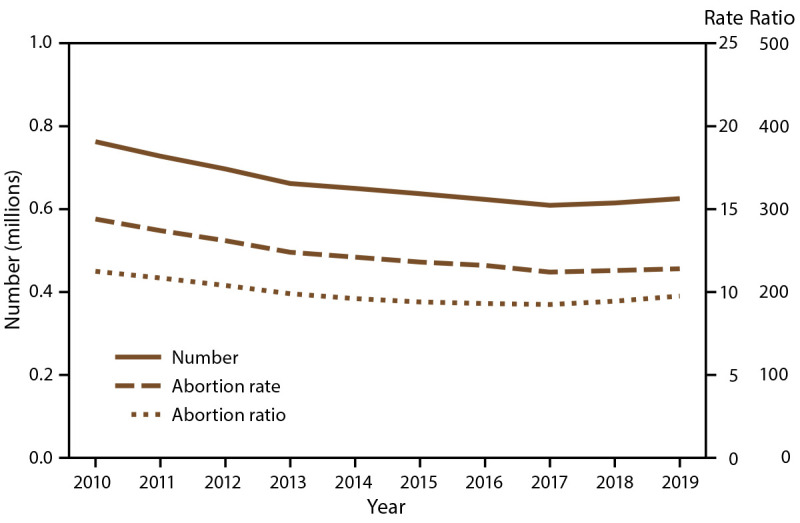
Number, rate,* and ratio† of abortions performed, by year – selected reporting areas,^§^ United States, 2011–2020 * Number of abortions per 1,000 women aged 15–44 years. ^†^ Number of abortions per 1,000 live births. ^§^ Data are for 48 reporting areas; excludes California, the District of Columbia, Maryland, and New Hampshire.

In 2020, there was a considerable range by reporting area of occurrence in abortion rates (from 0.1 to 23.0 abortions per 1,000 women aged 15–44 years in Missouri and the District of Columbia) and abortion ratios (from two to 498 abortions per 1,000 live births in Missouri and the District of Columbia) (Table 2). The percentage of abortions obtained by out-of-area residents also varied among reporting areas (from 0.5% in Arizona to 70.7% in the District of Columbia).

**TABLE 2:** Number, rate,* and ratio^†^ of reported abortions, by reporting area of occurrence and number of abortions obtained by out-of-area residents^§^ — United States, 2020^¶^

Area	Abortions reported by area of occurrence**			Abortions obtained by out-of-area residents
	No.	Rate	Ratio	No. (%)
Alabama	5,713	6.0	99	875 (15.3)
Alaska	1,206	8.4	127	8 (0.7)
Arizona	13,273	9.3	172	72 (0.5)
Arkansas	3,154	5.4	89	390 (12.4)
Colorado	9,869	8.3	160	1,283 (13.0)
Connecticut	9,115	13.6	272	456 (5.0)
Delaware	2,281	12.5	219	272 (11.9)
District of Columbia	4,416	23.0	498	3,123 (70.7)
Florida	74,868	19.1	357	3,988 (5.3)
Georgia	37,533	17.1	306	6,411 (17.1)
Hawaii	1,809	7.0	115	32 (1.8)
Idaho	1,680	4.8	78	102 (6.1)
Illinois	46,243	18.7	347	9,686 (20.9)
Indiana	7,756	5.9	99	384 (5.0)
Iowa	4,058	6.8	112	679 (16.7)
Kansas	7,526	13.4	219	3,901 (51.8)
Kentucky	4,104	4.8	79	617 (15.0)
Louisiana	7,473	8.1	130	1,240 (16.6)
Maine	2,064	8.8	179	115 (5.6)
Massachusetts	16,452	11.8	248	593 (3.6)
Michigan	29,669	15.8	285	1,620 (5.5)
Minnesota	10,349	9.5	163	971 (9.4)
Mississippi	3,559	6.1	100	360 (10.1)
Missouri	167	0.1	2	33 (19.8)
Montana	1,675	8.4	155	177 (10.6)
Nebraska	2,378	6.3	98	374 (15.7)
Nevada	8,633	14.1	257	471 (5.5)
New Jersey^††^	22,972	13.7	235	1,593 (6.9)
New Mexico	4,293	10.7	196	1,301 (30.3)
New York	63,142	16.5	302	3,670 (5.8)^§§^
New York City	37,523	20.9	388	3,195 (8.5)
New York State	25,619	12.6	227	2,469 (9.6)
North Carolina	30,004	14.4	257	5,117 (17.1)
North Dakota	1,174	7.9	117	338 (28.8)
Ohio	20,605	9.3	159	1,167 (5.7)
Oklahoma	3,797	4.9	80	598 (15.7)
Oregon	6,991	8.4	176	672 (9.6)
Pennsylvania	32,123	13.5	246	2,144 (6.7)
Rhode Island	2,611	12.6	258	424 (16.2)
South Carolina	5,468	5.5	98	278 (5.1)
South Dakota	125	0.8	11	19 (15.2)
Tennessee	11,243	8.4	143	—^¶¶^
Texas	55,132	9.0	150	1,183 (2.1)
Utah	2,362	3.3	52	118 (5.0)
Vermont	1,227	10.7	239	213 (17.4)
Virginia	15,604	9.2	165	1,067 (6.8)
Washington	16,909	11.0	204	852 (5.0)
West Virginia	1,001	3.2	58	152 (15.2)
Wisconsin	6,430	5.9	106	94 (1.5)
Wyoming	91	0.8	15	22 (24.2)
**Total**	**620,327**	**N/A**	**N/A**	**N/A**

**Abbreviation:** N/A = not applicable.

* Number of abortions per 1,000 women aged 15–44 years.

^†^ Number of abortions per 1,000 live births.

^§^ Additional details on the reporting area in which abortions were provided, cross-tabulated by the area of residence, are available at https://www.cdc.gov/reproductivehealth/data_stats/Abortion.htm.

^¶^ Data from 49 reporting areas; excludes three reporting areas (California, Maryland, and New Hampshire) that did not report.

** The total abortions include those with known and unknown residence status.

^††^ Reporting to the central health agency is not required. Data are requested from hospitals and licensed ambulatory care facilities only.

^§§^ Residents of New York State who had abortions in New York City and residents of New York City who had abortions in New York State were excluded from the number and percentage of abortions obtained by out-of-area residents in New York.

^¶¶^ Tennessee did not report data by residence; therefore, the percentage of abortions obtained in Tennessee by out-of-area residents cannot be calculated.

### Age Group, Race or Ethnicity, and Marital Status

Among the 48 areas that reported abortion numbers by women’s age for 2020, women in their 20s accounted for more than half of abortions (57.2%) (Table 3). Women aged 20–24 and 25–29 years accounted for the highest percentages of abortions (27.9% and 29.3%, respectively) and had the highest abortion rates (19.2 and 19.0 abortions per 1,000 women aged 20–24 and 25–29 years, respectively). By contrast, adolescents aged <15 years and women aged ≥40 years accounted for the lowest percentages of abortions (0.2% and 3.7%, respectively) and had the lowest abortion rates (0.4 and 2.6 abortions per 1,000 women aged <15 and ≥40 years, respectively). However, abortion ratios were highest among adolescents (859 and 363 abortions per 1,000 live births among those aged <15 years and 15–19 years, respectively) and lowest among women aged 25–39 years (205, 136, and 144 abortions per 1,000 live births among those aged 25–29, 30–34, and 35–39 years, respectively).

**TABLE 3:** Number of reported abortions, by known age group and reporting area of occurrence — selected reporting areas,* United States, 2020

Area	Age group (yrs)							Total abortions reported by known age
	<15	15–19	20–24	25–29	30–34	35–39	≥40	
	No. (%)^†^	No. (%)	No. (%)	No. (%)	No. (%)	No. (%)	No. (%)	No. (% of all reported abortions)^§^
Alabama	21 (0.4)	483 (8.5)	1,746 (30.6)	1,768 (31.0)	1,019 (17.9)	533 (9.3)	137 (2.4)	**5,707 (99.9)**
Alaska	7 (0.6)	121 (10.0)	361 (29.9)	319 (26.5)	238 (19.7)	121 (10.0)	39 (3.2)	**1,206 (100.0)**
Arizona	24 (0.2)	1,202 (9.1)	4,033 (30.4)	3,656 (27.5)	2,519 (19.0)	1,371 (10.3)	468 (3.5)	**13,273 (100.0)**
Arkansas	11 (0.3)	293 (9.3)	965 (30.6)	961 (30.5)	556 (17.7)	279 (8.9)	84 (2.7)	**3,149 (99.8)**
Colorado	28 (0.3)	929 (9.4)	2,897 (29.4)	2,812 (28.5)	1,890 (19.2)	953 (9.7)	357 (3.6)	**9,866 (100.0)**
Connecticut	15 (0.2)	770 (8.5)	2,575 (28.3)	2,556 (28.1)	1,849 (20.3)	1,017 (11.2)	325 (3.6)	**9,107 (99.9)**
Delaware	8 (0.4)	221 (9.7)	656 (28.8)	646 (28.3)	424 (18.6)	248 (10.9)	78 (3.4)	**2,281 (100.0)**
District of Columbia	11 (0.2)	407 (9.2)	1,273 (28.8)	1,366 (31.0)	819 (18.6)	407 (9.2)	130 (2.9)	**4,413 (99.9)**
Florida	124 (0.2)	5,157 (6.9)	20,017 (26.8)	21,866 (29.3)	15,876 (21.2)	8,613 (11.5)	3,087 (4.1)	**74,740 (99.8)**
Georgia	66 (0.2)	2,666 (7.1)	10,444 (27.8)	11,572 (30.8)	7,674 (20.4)	3,814 (10.2)	1,297 (3.5)	**37,533 (100.0)**
Hawaii	—^¶^	157 (8.7)	501 (27.7)	499 (27.6)	334 (18.5)	218 (12.1)	—^¶^	**1,809 (100.0)**
Idaho	9 (0.5)	195 (11.6)	546 (32.5)	423 (25.2)	279 (16.6)	174 (10.4)	54 (3.2)	**1,680 (100.0)**
Illinois	88 (0.2)	3,775 (8.2)	13,022 (28.3)	14,343 (31.2)	8,809 (19.1)	4,554 (9.9)	1,438 (3.1)	**46,029 (99.5)**
Indiana	16 (0.2)	699 (9.0)	2,367 (30.5)	2,232 (28.8)	1,417 (18.3)	771 (9.9)	254 (3.3)	**7,756 (100.0)**
Iowa	14 (0.3)	409 (10.1)	1,177 (29.0)	1,133 (27.9)	732 (18.0)	458 (11.3)	135 (3.3)	**4,058 (100.0)**
Kansas	20 (0.3)	694 (9.2)	2,355 (31.3)	2,154 (28.6)	1,288 (17.1)	761 (10.1)	254 (3.4)	**7,526 (100.0)**
Kentucky	13 (0.3)	353 (8.6)	1,192 (29.0)	1,229 (29.9)	779 (19.0)	399 (9.7)	139 (3.4)	**4,104 (100.0)**
Louisiana	22 (0.3)	634 (8.5)	2,098 (28.1)	2,257 (30.2)	1,446 (19.4)	762 (10.2)	253 (3.4)	**7,472 (100.0)**
Maine	7 (0.3)	164 (8.0)	553 (26.8)	584 (28.3)	419 (20.3)	267 (13.0)	67 (3.3)	**2,061 (99.9)**
Massachusetts	19 (0.1)	1,118 (6.8)	4,216 (25.7)	4,430 (27.0)	3,643 (22.2)	2,192 (13.4)	761 (4.6)	**16,379 (99.6)**
Michigan	74 (0.3)	2,360 (8.0)	8,492 (28.8)	9,275 (31.4)	5,810 (19.7)	2,625 (8.9)	881 (3.0)	**29,517 (99.5)**
Minnesota	28 (0.3)	857 (8.3)	2,766 (26.8)	2,938 (28.4)	2,142 (20.7)	1,186 (11.5)	419 (4.1)	**10,336 (99.9)**
Mississippi	12 (0.3)	296 (8.3)	1,077 (30.3)	1,145 (32.2)	636 (17.9)	315 (8.9)	78 (2.2)	**3,559 (100.0)**
Missouri	—^¶^	8 (4.8)	36 (21.6)	40 (24.0)	39 (23.4)	35 (21.0)	—^¶^	**167 (100.0)**
Montana	6 (0.4)	200 (11.9)	460 (27.5)	445 (26.6)	304 (18.1)	191 (11.4)	69 (4.1)	**1,675 (100.0)**
Nebraska	9 (0.4)	245 (10.3)	711 (29.9)	671 (28.2)	423 (17.8)	217 (9.1)	102 (4.3)	**2,378 (100.0)**
Nevada	22 (0.3)	795 (9.5)	2,243 (26.8)	2,308 (27.6)	1,709 (20.4)	955 (11.4)	331 (4.0)	**8,363 (96.9)**
New Jersey**	42 (0.2)	1,983 (8.6)	5,929 (25.8)	6,625 (28.8)	4,721 (20.6)	2,634 (11.5)	1,033 (4.5)	**22,967 (100.0)**
New Mexico	23 (0.6)	491 (11.9)	1,296 (31.5)	1,060 (25.8)	707 (17.2)	411 (10.0)	123 (3.0)	**4,111 (95.8)**
New York	126 (0.2)	5,338 (8.5)	16,336 (25.9)	17,895 (28.4)	13,126 (20.8)	7,396 (11.7)	2,745 (4.4)	**62,962 (99.7)**
New York City	58 (0.2)	2,931 (7.8)	9,339 (24.9)	10,729 (28.6)	8,114 (21.6)	4,562 (12.2)	1,790 (4.8)	**37,523 (100.0)**
New York State	68 (0.3)	2,407 (9.5)	6,997 (27.5)	7,166 (28.2)	5,012 (19.7)	2,834 (11.1)	955 (3.8)	**25,439 (99.3)**
North Carolina	56 (0.2)	2,201 (7.6)	8,204 (28.3)	8,818 (30.4)	5,832 (20.1)	2,950 (10.2)	906 (3.1)	**28,967 (96.5)**
North Dakota	—^¶^	109 (9.3)	352 (30.0)	336 (28.6)	225 (19.2)	109 (9.3)	—^¶^	**1,174 (100.0)**
Ohio	52 (0.3)	1,702 (8.3)	5,915 (28.7)	6,285 (30.5)	3,945 (19.1)	1,993 (9.7)	713 (3.5)	**20,605 (100.0)**
Oklahoma	21 (0.6)	373 (9.8)	1,208 (31.9)	1,061 (28.0)	687 (18.1)	318 (8.4)	121 (3.2)	**3,789 (99.8)**
Oregon	20 (0.3)	653 (9.3)	2,089 (29.9)	1,819 (26.0)	1,303 (18.6)	775 (11.1)	332 (4.7)	**6,991 (100.0)**
Pennsylvania	102 (0.3)	2,437 (7.6)	8,627 (26.9)	9,841 (30.6)	6,682 (20.8)	3,344 (10.4)	1,090 (3.4)	**32,123 (100.0)**
Rhode Island	—^¶^	203 (7.8)	770 (29.5)	755 (28.9)	519 (19.9)	265 (10.2)	—^¶^	**2,610 (100.0)**
South Carolina	14 (0.3)	495 (9.1)	1,556 (28.5)	1,590 (29.1)	1,050 (19.2)	581 (10.6)	182 (3.3)	**5,468 (100.0)**
South Dakota	—^¶^	9 (7.2)	38 (30.4)	37 (29.6)	28 (22.4)	9 (7.2)	—^¶^	**125 (100.0)**
Texas	107 (0.2)	4,611 (8.4)	16,276 (29.5)	15,793 (28.6)	10,486 (19.0)	5,900 (10.7)	1,959 (3.6)	**55,132 (100.0)**
Utah	6 (0.3)	276 (11.7)	760 (32.2)	583 (24.7)	402 (17.1)	234 (9.9)	96 (4.1)	**2,357 (99.8)**
Vermont	6 (0.5)	94 (7.7)	330 (26.9)	329 (26.8)	255 (20.8)	157 (12.8)	56 (4.6)	**1,227 (100.0)**
Virginia	21 (0.1)	1,065 (6.8)	4,130 (26.5)	4,576 (29.3)	3,271 (21.0)	1,904 (12.2)	631 (4.0)	**15,598 (100.0)**
Washington	36 (0.2)	1,597 (9.5)	4,698 (27.8)	4,473 (26.5)	3,347 (19.8)	2,032 (12.0)	698 (4.1)	**16,881 (99.8)**
West Virginia	—^¶^	90 (9.0)	306 (30.6)	317 (31.7)	170 (17.0)	85 (8.5)	—^¶^	**1,001 (100.0)**
Wisconsin	14 (0.2)	627 (9.8)	1,908 (29.7)	1,864 (29.0)	1,196 (18.6)	617 (9.6)	204 (3.2)	**6,430 (100.0)**
Wyoming	0 (—)	7 (7.7)	22 (24.2)	24 (26.4)	21 (23.1)	10 (11.0)	7 (7.7)	**91 (100.0)**
**Total**	**1,333 (0.2)**	**49,569 (8.2)**	**169,529 (27.9)**	**177,709 (29.3)**	**121,046 (19.9)**	**65,160 (10.7)**	**22,407 (3.7)**	**606,753 (99.6)** ^††^
**Abortion rate** ^§§^	**0.4**	**5.8**	**19.2**	**19.0**	**13.0**	**7.2**	**2.6**	**N/A**
**Abortion ratio** ^¶¶^	**859**	**363**	**296**	**205**	**136**	**144**	**219**	**N/A**

**Abbreviation:** N/A = not applicable.

* Data from 48 reporting areas; excludes four reporting areas (California, Maryland, New Hampshire, and Tennessee) that did not report, did not report by age, or did not meet reporting standards.

^†^ Percentages for the individual component categories might not add to 100% because of rounding.

^§^ Percentage is calculated as the number of abortions reported by known age divided by the sum of abortions reported by known and unknown age. Values ≥99.95% are rounded to 100.0%.

^¶^ Cells with a numerical value in the range of 1–4 and cells that would allow for calculation of these small values have been suppressed.

** Reporting to the central health agency is not required. Data are requested from hospitals and licensed ambulatory care facilities only.

^††^ Percentage based on a total of 609,084 abortions reported among the areas that met reporting standards for age.

^§§^ Number of abortions obtained by women in a given age group per 1,000 women in that same age group. Adolescents aged 13–14 years were used as the denominator for the group of adolescents aged <15 years, and women aged 40–44 years were used as the denominator for the group of women aged ≥40 years. For the total abortion rate only, abortions for women of unknown age were distributed according to the distribution of abortions among women of known age.

^¶¶^ Number of abortions obtained by women in a given age group per 1,000 live births to women in that same age group. For the total abortion ratio only, abortions for women of unknown age were distributed according to the distribution of abortions among women of known age.

Among the 43 reporting areas that provided data each year by women’s age for 2011–2020, this pattern across age groups was stable, with the highest percentages of abortions and the highest abortion rates occurring among women aged 20–29 years and the lowest percentages of abortions and lowest abortion rates occurring among adolescents aged <15 years and women aged ≥40 years (Table 4). From 2011 to 2020, abortion rates decreased among all age groups, although the decreases for adolescents (56% and 49% for adolescents aged <15 and 15–19 years, respectively) were greater than the decreases for women aged ≥20 years. From 2019 to 2020, abortion rates decreased or did not change for all age groups. During 2011–2020, abortion ratios decreased for all age groups, except among adolescents aged 15–19 years and women aged 25–29 years for whom abortion ratios increased. The decrease in abortion ratio was highest among women aged ≥40 years. From 2019 to 2020, abortion ratios decreased for adolescents aged <15 years and women aged ≥35 years and increased for women aged 15–34 years.

**TABLE 4:** Percentage, rate,* and ratio^†^ of reported abortions, by known age group and year — selected reporting areas,^§^ United States, 2011–2020

Age group (yrs)	Year										% Change	
	2011	2012	2013	2014	2015	2016	2017	2018	2019	2020	2019 to 2020	2011 to 2020
**Reported abortions by known age (%)**												
<15	0.4	0.4	0.3	0.3	0.3	0.3	0.2	0.2	0.2	0.2	0.0	−50.0
15–19	13.5	12.2	11.4	10.4	9.8	9.4	9.1	8.8	8.7	8.3	−4.6	−38.5
20–24	32.9	32.7	32.7	32.1	31.1	30.0	29.3	28.5	27.8	28.1	1.1	−14.6
25–29	24.9	25.3	25.9	26.7	27.6	28.5	29.0	29.3	29.3	29.3	0.0	17.7
30–34	15.8	16.4	16.9	17.2	17.7	18.0	18.3	18.8	19.4	19.8	2.1	25.3
35–39	8.9	9.1	9.2	9.7	10.0	10.3	10.5	10.7	10.9	10.6	−2.8	19.1
≥40	3.6	3.8	3.6	3.6	3.6	3.6	3.6	3.6	3.7	3.6	−2.7	0.0
**Abortion rate**												
<15	0.9	0.8	0.6	0.5	0.5	0.4	0.4	0.4	0.4	0.4	0.0	−55.6
15–19	10.5	9.2	8.2	7.3	6.7	6.3	5.9	5.8	5.9	5.5	−6.8	−47.6
20–24	25.0	23.3	21.9	21.0	20.0	19.1	18.4	18.3	18.2	18.2	0.0	−27.2
25–29	19.5	18.9	18.2	18.2	18.0	17.8	17.4	17.7	17.9	17.9	0.0	−8.2
30–34	12.7	12.4	11.9	11.7	11.8	11.7	11.5	11.9	12.4	12.2	−1.6	−3.9
35–39	7.5	7.4	7.0	7.1	7.0	6.9	6.8	6.9	7.0	6.8	−2.9	−9.3
≥40	2.8	2.8	2.6	2.6	2.5	2.5	2.5	2.5	2.6	2.5	−3.8	−10.7
**Abortion ratio**												
<15	846	801	791	744	706	743	799	878	875	816	−6.7	−3.5
15–19	328	306	302	294	293	299	306	323	338	346	−2.4	5.5
20–24	286	273	264	258	253	252	252	259	264	278	5.3	−2.8
25–29	178	174	169	167	168	170	172	179	185	191	3.2	7.3
30–34	132	128	121	116	116	113	115	119	125	127	1.6	−3.8
35–39	164	157	147	144	140	136	134	135	138	135	−2.2	−17.7
≥40	274	269	244	240	228	219	211	207	214	206	−3.7	−24.8
Total no.^¶,^**	**624,711**	**595,784**	**565,418**	**553,940**	**542,330**	**531,735**	**517,522**	**522,703**	**529,942**	**524,221**	**N/A**	**N/A**

**Abbreviation:** N/A = not applicable.

* Number of abortions obtained by women in a given age group per 1,000 women in that same age group. Adolescents aged 13–14 years were used as the denominator for the group of adolescents aged <15 years, and women aged 40–44 years were used as the denominator for the group of women aged ≥40 years. Abortions for women of unknown age were distributed according to the distribution of abortions among women of known age.

^†^ Number of abortions obtained by women in a given age group per 1,000 live births to women in that same age group. Abortions for women of unknown age were distributed according to the distribution of abortions among women of known age.

^§^ Data from 43 reporting areas; excludes nine reporting areas (California, the District of Columbia, Florida, Maine, Maryland, New Hampshire, Tennessee, Vermont, and Wyoming) that did not report, did not report by age, or did not meet reporting standards for ≥1 year.

^¶^ By year, the total number of abortions represent 99.5%–99.7% of all abortions reported to CDC among the areas that met reporting standards for age during 2011–2020; reporting standards for age were applied to abortions for residents of Illinois only during 2011−2019.

** The total number is different than previously reported because the total by known age is presented, and data for out-of-area residents were subsequently added for Wisconsin.

Among the 46 areas that reported age by individual year among adolescents for 2020, adolescents aged 18–19 years accounted for highest percentage (71.1%) of adolescent abortions and had the highest abortion rates (8.3 and 11.9 abortions per 1,000 adolescents aged 18 and 19 years, respectively) (Table 5). Adolescents aged <15 years accounted for the lowest percentage of adolescent abortions (2.7%) and had the lowest abortion rate (0.4 abortions per 1,000 adolescents aged 13–14 years). The abortion ratio for adolescents was highest among adolescents aged <15 years (828 abortions per 1,000 live births) and was lowest among adolescents aged 17–19 years (343, 371, and 313 abortions per 1,000 live births among adolescents aged 17, 18, and 19 years, respectively).

**TABLE 5:** Number of reported abortions among adolescents, by known age and reporting area of occurrence — selected reporting areas,* United States, 2020

Area	Age (yrs)						Total no.
	<15	15	16	17	18	19	
	No. (%)^†^	No. (%)	No. (%)	No. (%)	No. (%)	No. (%)	
Alabama	21 (4.2)	29 (5.8)	48 (9.5)	58 (11.5)	146 (29.0)	202 (40.1)	**504**
Alaska	7 (5.5)	5 (3.9)	16 (12.5)	23 (18.0)	15 (11.7)	62 (48.4)	**128**
Arizona	24 (2.0)	47 (3.8)	71 (5.8)	134 (10.9)	362 (29.5)	588 (48.0)	**1,226**
Arkansas	11 (3.6)	15 (4.9)	21 (6.9)	41 (13.5)	81 (26.6)	135 (44.4)	**304**
Colorado	28 (2.9)	49 (5.1)	78 (8.2)	137 (14.3)	270 (28.2)	395 (41.3)	**957**
Delaware	—^§^	—^§^	—^§^	—^§^	—^§^	—^§^	**—^§^**
District of Columbia	11 (2.6)	15 (3.6)	52 (12.4)	81 (19.4)	102 (24.4)	157 (37.6)	**418**
Florida	124 (2.3)	186 (3.5)	372 (7.0)	633 (12.0)	1,489 (28.2)	2,477 (46.9)	**5,281**
Georgia	66 (2.4)	94 (3.4)	211 (7.7)	317 (11.6)	814 (29.8)	1,230 (45.0)	**2,732**
Hawaii	—^§^	—^§^	—^§^	—^§^	—^§^	—^§^	**—^§^**
Idaho	9 (4.4)	7 (3.4)	20 (9.8)	17 (8.3)	66 (32.4)	85 (41.7)	**204**
Indiana	16 (2.2)	39 (5.5)	60 (8.4)	82 (11.5)	199 (27.8)	319 (44.6)	**715**
Iowa	14 (3.3)	21 (5.0)	45 (10.6)	60 (14.2)	107 (25.3)	176 (41.6)	**423**
Kansas	20 (2.8)	29 (4.1)	65 (9.1)	87 (12.2)	222 (31.1)	291 (40.8)	**714**
Kentucky	13 (3.6)	23 (6.3)	41 (11.2)	49 (13.4)	91 (24.9)	149 (40.7)	**366**
Louisiana	22 (3.4)	42 (6.4)	64 (9.8)	90 (13.7)	194 (29.6)	244 (37.2)	**656**
Maine	7 (4.1)	6 (3.5)	25 (14.6)	34 (19.9)	47 (27.5)	52 (30.4)	**171**
Massachusetts	19 (1.7)	46 (4.0)	88 (7.7)	150 (13.2)	296 (26.0)	538 (47.3)	**1,137**
Michigan	74 (3.0)	104 (4.3)	198 (8.1)	348 (14.3)	696 (28.6)	1,014 (41.7)	**2,434**
Minnesota	28 (3.2)	39 (4.4)	65 (7.3)	132 (14.9)	261 (29.5)	360 (40.7)	**885**
Mississippi	12 (3.9)	18 (5.8)	25 (8.1)	36 (11.7)	87 (28.2)	130 (42.2)	**308**
Missouri	—^§^	—^§^	—^§^	—^§^	—^§^	—^§^	**—^§^**
Montana	6 (2.9)	9 (4.4)	16 (7.8)	39 (18.9)	61 (29.6)	75 (36.4)	**206**
Nebraska	9 (3.5)	16 (6.3)	18 (7.1)	30 (11.8)	86 (33.9)	95 (37.4)	**254**
Nevada	22 (2.7)	39 (4.8)	66 (8.1)	108 (13.2)	253 (31.0)	329 (40.3)	**817**
New Jersey^¶^	42 (2.1)	77 (3.8)	186 (9.2)	398 (19.7)	558 (27.6)	764 (37.7)	**2,025**
New Mexico	23 (4.5)	30 (5.8)	42 (8.2)	100 (19.5)	125 (24.3)	194 (37.7)	**514**
New York	126 (2.3)	248 (4.5)	459 (8.4)	914 (16.7)	1,515 (27.7)	2,202 (40.3)	**5,464**
New York City	58 (1.9)	135 (4.5)	233 (7.8)	502 (16.8)	787 (26.3)	1,274 (42.6)	**2,989**
New York State	68 (2.7)	113 (4.6)	226 (9.1)	412 (16.6)	728 (29.4)	928 (37.5)	**2,475**
North Carolina	56 (2.5)	94 (4.2)	186 (8.2)	252 (11.2)	665 (29.5)	1,004 (44.5)	**2,257**
North Dakota	—^§^	—^§^	—^§^	—^§^	—^§^	—^§^	**—^§^**
Ohio	52 (3.0)	100 (5.7)	133 (7.6)	236 (13.5)	524 (29.9)	709 (40.4)	**1,754**
Oklahoma	21 (5.3)	9 (2.3)	32 (8.1)	40 (10.2)	118 (29.9)	174 (44.2)	**394**
Oregon	20 (3.0)	32 (4.8)	53 (7.9)	78 (11.6)	210 (31.2)	280 (41.6)	**673**
Pennsylvania	102 (4.0)	130 (5.1)	212 (8.3)	311 (12.2)	713 (28.1)	1,071 (42.2)	**2,539**
Rhode Island	—^§^	—^§^	—^§^	—^§^	—^§^	—^§^	**—^§^**
South Carolina	14 (2.8)	16 (3.1)	42 (8.3)	118 (23.2)	130 (25.5)	189 (37.1)	**509**
South Dakota	—^§^	—^§^	—^§^	—^§^	—^§^	—^§^	**—^§^**
Texas	107 (2.3)	179 (3.8)	388 (8.2)	603 (12.8)	1,339 (28.4)	2,102 (44.6)	**4,718**
Utah	6 (2.1)	13 (4.6)	16 (5.7)	27 (9.6)	85 (30.1)	135 (47.9)	**282**
Vermont	—^§^	—^§^	—^§^	—^§^	—^§^	—^§^	**—^§^**
Virginia	21 (1.9)	51 (4.7)	86 (7.9)	136 (12.5)	321 (29.6)	471 (43.4)	**1,086**
Washington	36 (2.2)	77 (4.7)	130 (8.0)	238 (14.6)	465 (28.5)	687 (42.1)	**1,633**
West Virginia	—^§^	—^§^	—^§^	—^§^	—^§^	—^§^	**—^§^**
Wisconsin**	14 (2.2)	34 (5.4)	54 (8.6)	83 (13.2)	199 (31.6)	246 (39.0)	**630**
Wyoming	—^§^	—^§^	—^§^	—^§^	—^§^	—^§^	**—^§^**
**Total**	**1,230 (2.7)**	**1,997 (4.3)**	**3,765 (8.1)**	**6,354 (13.7)**	**13,198 (28.5)**	**19,699 (42.6)**	**46,243**
**Abortion rate^††^**	**0.4**	**1.2**	**2.3**	**4.0**	**8.3**	**11.9**	**N/A**
**Abortion ratio^§§^**	**828**	**517**	**404**	**343**	**371**	**313**	**N/A**

**Abbreviation:** N/A = not applicable.

* Data from 46 reporting areas; excludes six reporting areas (California, Connecticut, Illinois, Maryland, New Hampshire, and Tennessee) that did not report, did not report age among adolescents by individual year, or did not meet reporting standards.

^†^ Percentages for the individual component categories might not add to 100% because of rounding.

^§^ Cells with a numerical value in the range of 1–4 and cells that would allow for calculation of these small values have been suppressed.

^¶^ Reporting to the central health agency is not required. Data are requested from hospitals and licensed ambulatory care facilities only.

** Includes residents only.

^††^ Number of abortions obtained by women in a given age group per 1,000 women in that same age group. Adolescents aged 13–14 years were used as the denominator for the group of adolescents aged <15 years. For the total abortion rate only, abortions for women of unknown age were distributed according to the distribution of abortions among women of known age.

^§§^ Number of abortions obtained by women in a given age group per 1,000 live births to women in that same age group. For the total abortion ratio only, abortions for women of unknown age were distributed according to the distribution of abortions among women of known age.

Among the 30 areas that reported race by ethnicity data for 2020, non-Hispanic White women (White) and non-Hispanic Black women (Black) accounted for the highest percentages of all abortions (32.7% and 39.2%, respectively), and Hispanic women and non-Hispanic women in the other race category accounted for lower percentages (21.1% and 7.0%, respectively) (Table 6). White women had the lowest abortion rate (6.2 abortions per 1,000 women aged 15–44 years) and ratio (118 abortions per 1,000 live births), and Black women had the highest abortion rate (24.4 abortions per 1,000 women aged 15–44 years) and ratio (426 abortions per 1,000 live births).

**TABLE 6:** Number of reported abortions, by known race or ethnicity and reporting area of occurrence — selected reporting areas,* United States, 2020

Area	Non-Hispanic			Hispanic	Total abortions reported by known race or ethnicity
	White	Black	Other^†^		
	No. (%)^§^	No. (%)	No. (%)	No. (%)	No. (% of all reported abortions)^¶^
Alabama	1,485 (26.0)	3,769 (66.1)	116 (2.0)	334 (5.9)	**5,704 (99.8)**
Alaska	530 (47.2)	77 (6.9)	459 (40.9)	56 (5.0)	**1,122 (93.0)**
Arizona	4,705 (36.4)	1,606 (12.4)	1,244 (9.6)	5,374 (41.6)	**12,929 (97.4)**
Arkansas	1,145 (36.5)	1,704 (54.4)	103 (3.3)	182 (5.8)	**3,134 (99.4)**
Connecticut	3,037 (33.8)	2,452 (27.3)	551 (6.1)	2,951 (32.8)	**8,991 (98.6)**
Delaware	858 (37.6)	1,065 (46.7)	48 (2.1)	310 (13.6)	**2,281 (100.0)**
District of Columbia	651 (16.2)	31 (0.8)	2,610 (64.9)	727 (18.1)	**4,019 (91.0)**
Florida	20,706 (29.6)	26,213 (37.5)	2,684 (3.8)	20,299 (29.0)	**69,902 (93.4)**
Georgia	6,888 (19.5)	23,534 (66.5)	1,689 (4.8)	3,256 (9.2)	**35,367 (94.2)**
Idaho	1,107 (70.0)	38 (2.4)	67 (4.2)	369 (23.3)	**1,581 (94.1)**
Indiana	3,603 (47.2)	2,648 (34.7)	557 (7.3)	832 (10.9)	**7,640 (98.5)**
Kansas	3,889 (51.8)	1,913 (25.5)	613 (8.2)	1,097 (14.6)	**7,512 (99.8)**
Kentucky	2,192 (53.7)	1,412 (34.6)	170 (4.2)	310 (7.6)	**4,084 (99.5)**
Michigan	10,498 (36.9)	15,470 (54.4)	1,418 (5.0)	1,028 (3.6)	**28,414 (95.8)**
Minnesota	4,646 (49.7)	2,853 (30.5)	1,229 (13.2)	616 (6.6)	**9,344 (90.3)**
Mississippi	624 (17.6)	2,749 (77.3)	79 (2.2)	103 (2.9)	**3,555 (99.9)**
Missouri	87 (56.1)	49 (31.6)	12 (7.7)	7 (4.5)	**155 (92.8)**
Montana	1,381 (82.4)	28 (1.7)	165 (9.9)	101 (6.0)	**1,675 (100.0)**
Nevada	2,766 (34.4)	1,753 (21.8)	1,000 (12.4)	2,529 (31.4)	**8,048 (93.2)**
New Mexico	966 (25.6)	218 (5.8)	393 (10.4)	2,190 (58.1)	**3,767 (87.7)**
North Carolina	7,871 (27.9)	14,738 (52.3)	2,049 (7.3)	3,548 (12.6)	**28,206 (94.0)**
Oregon	4,143 (61.8)	395 (5.9)	738 (11.0)	1,430 (21.3)	**6,706 (95.9)**
South Carolina	2,404 (44.0)	2,363 (43.2)	244 (4.5)	457 (8.4)	**5,468 (100.0)**
South Dakota	80 (64.0)	—**	—**	6 (4.8)	**125 (100.0)**
Texas^††^	14,473 (26.6)	16,393 (30.1)	3,450 (6.3)	20,152 (37.0)	**54,468 (98.8)**
Utah	1,326 (56.4)	106 (4.5)	182 (7.7)	736 (31.3)	**2,350 (99.5)**
Vermont	1,054 (87.7)	56 (4.7)	57 (4.7)	35 (2.9)	**1,202 (98.0)**
Virginia	4,895 (33.9)	6,746 (46.7)	1,457 (10.1)	1,356 (9.4)	**14,454 (92.6)**
West Virginia	836 (83.5)	143 (14.3)	17 (1.7)	5 (0.5)	**1,001 (100.0)**
Wyoming	58 (68.2)	—**	—**	24 (28.2)	**85 (93.4)**
**Total**	**108,904 (32.7)**	**130,538 (39.2)**	**23,427 (7.0)**	**70,420 (21.1)**	**333,289 (95.5)** ^§§^
**Abortion rate** ^¶¶^	**6.2**	**24.4**	**12.7**	**11.4**	**N/A**
Abortion ratio***	**118**	**426**	**186**	**173**	**N/A**

**Abbreviation:** N/A = not applicable.

* Data from 30 reporting areas; excludes 22 reporting areas (California, Colorado, Hawaii, Illinois, Iowa, Louisiana, Maine, Maryland, Massachusetts, Nebraska, New Hampshire, New Jersey, New York City, New York State, North Dakota, Ohio, Oklahoma, Pennsylvania, Rhode Island, Tennessee, Washington, and Wisconsin) that did not report, did not report by race or ethnicity, or did not meet reporting standards.

^†^ Including Asian (Indian, Chinese, Filipino, Japanese, Korean, Vietnamese, or other Asian), Pacific Islander (Native Hawaiian, Guamanian or Chamorro, Samoan, or other Pacific Islander), other races, and multiple races.

^§^ Percentages for the individual component categories might not add to 100% because of rounding.

^¶^ Percentage is calculated as the number of abortions reported by known race or ethnicity divided by the sum of abortions reported by known and unknown race or ethnicity. Values ≥99.95% are rounded to 100.0%.

** Cells with a numerical value in the range of 1–4 and cells that would allow for calculation of these small values have been suppressed.

^††^ Reporting form contains only one question for race or ethnicity; therefore, abortions reported for women of White, Black, and other races (Asian and Native American) are not explicitly identified as non-Hispanic.

^§§^ Percentage based on a total of 348,975 abortions reported among the areas that met reporting standards for race and ethnicity.

^¶¶^ Number of abortions obtained by women in a given racial or ethnic group per 1,000 women aged 15–44 years in that same racial or ethnic group. For the total abortion rate only, abortions for women of unknown race and ethnicity were distributed according to the distribution of abortions among women of known race or ethnicity.

*** Number of abortions obtained by women in a given racial or ethnic group per 1,000 live births to women in that same racial or ethnic group. For the total abortion ratio only, abortions for women of unknown race or ethnicity were distributed according to the distribution of abortions among women of known race or ethnicity.

Among the 40 areas that reported by marital status for 2020, 13.7% of women who obtained an abortion were married, and 86.3% were unmarried (Table 7). The abortion ratio was 46 abortions per 1,000 live births for married women and 412 abortions per 1,000 live births for unmarried women.

**TABLE 7:** Number of reported abortions, by known marital status and reporting area of occurrence — selected reporting areas,* United States, 2020

Area	Marital status		Total abortions reported by known marital status
	Married	Unmarried	
	No. (%)^†^	No. (%)	No. (% of all reported abortions)^§^
Alabama	560 (9.8)	5,142 (90.2)	**5,702 (99.8)**
Alaska	253 (22.0)	898 (78.0)	**1,151 (95.4)**
Arizona	1,944 (14.6)	11,329 (85.4)	**13,273 (100.0)**
Arkansas	322 (10.3)	2,805 (89.7)	**3,127 (99.1)**
Colorado	1,645 (18.0)	7,469 (82.0)	**9,114 (92.3)**
Delaware	289 (12.7)	1,992 (87.3)	**2,281 (100.0)**
Florida	9,800 (15.2)	54,764 (84.8)	**64,564 (86.2)**
Georgia	4,112 (11.6)	31,188 (88.4)	**35,300 (94.1)**
Idaho	349 (21.7)	1,258 (78.3)	**1,607 (95.7)**
Illinois	4,037 (9.1)	40,475 (90.9)	**44,512 (96.3)**
Indiana	1,163 (15.0)	6,592 (85.0)	**7,755 (100.0)**
Iowa	601 (14.8)	3,451 (85.2)	**4,052 (99.9)**
Kansas	1,099 (14.7)	6,380 (85.3)	**7,479 (99.4)**
Kentucky	523 (12.7)	3,581 (87.3)	**4,104 (100.0)**
Louisiana	701 (9.5)	6,641 (90.5)	**7,342 (98.2)**
Maine	296 (16.3)	1,524 (83.7)	**1,820 (88.2)**
Michigan	2,957 (10.5)	25,198 (89.5)	**28,155 (94.9)**
Minnesota	1,586 (15.9)	8,371 (84.1)	**9,957 (96.2)**
Mississippi	285 (8.1)	3,255 (91.9)	**3,540 (99.5)**
Missouri	63 (38.9)	99 (61.1)	**162 (97.0)**
Montana	269 (16.1)	1,405 (83.9)	**1,674 (99.9)**
Nebraska	315 (13.5)	2,010 (86.5)	**2,325 (97.8)**
New Jersey^¶^	2,910 (13.0)	19,523 (87.0)	**22,433 (97.7)**
New Mexico	621 (15.3)	3,439 (84.7)	**4,060 (94.6)**
New York City	5,661 (17.3)	27,126 (82.7)	**32,787 (87.4)**
North Carolina	3,841 (13.9)	23,756 (86.1)	**27,597 (92.0)**
North Dakota	183 (15.6)	991 (84.4)	**1,174 (100.0)**
Ohio	2,658 (13.9)	16,466 (86.1)	**19,124 (92.8)**
Oklahoma	699 (18.4)	3,090 (81.6)	**3,789 (99.8)**
Oregon	1,298 (21.3)	4,804 (78.7)	**6,102 (87.3)**
Pennsylvania	3,755 (11.7)	28,281 (88.3)	**32,036 (99.7)**
Rhode Island	325 (12.7)	2,242 (87.3)	**2,567 (98.3)**
South Carolina	724 (13.2)	4,743 (86.8)	**5,467 (100.0)**
South Dakota	18 (14.4)	107 (85.6)	**125 (100.0)**
Texas	8,776 (15.9)	46,346 (84.1)	**55,122 (100.0)**
Utah	505 (21.5)	1,840 (78.5)	**2,345 (99.3)**
Vermont	242 (22.2)	846 (77.8)	**1,088 (88.7)**
Virginia**	1,800 (11.5)	13,804 (88.5)	**15,604 (100.0)**
West Virginia	160 (16.0)	840 (84.0)	**1,000 (99.9)**
Wisconsin	792 (12.4)	5,578 (87.6)	**6,370 (99.1)**
**Total**	**68,137 (13.7)**	**429,649 (86.3)**	**497,786 (94.6)** ^††^
**Abortion ratio** ^§§^	**46**	**412**	**N/A**

**Abbreviation:** N/A = not applicable.

* Data from 40 reporting areas excludes 12 reporting areas (California, Connecticut, the District of Columbia, Hawaii, Maryland, Massachusetts, Nevada, New Hampshire, New York State, Tennessee, Washington, and Wyoming) that did not report, did not report by marital status, or did not meet reporting standards.

^†^ Percentages for the individual component categories might not add to 100% because of rounding.

^§^ Percentage is calculated as the number of abortions reported by known marital status divided by the sum of abortions reported by known and unknown marital status. Values ≥99.95% are rounded to 100.0%.

^¶^ Reporting to the central health agency is not required. Data are requested from hospitals and licensed ambulatory care facilities only.

** Recorded as patient married or not married to father.

^††^ Percentage based on a total of 526,040 abortions reported among the areas that met reporting standards for marital status.

^§§^ Number of abortions obtained by marital status per 1,000 live births to women of the same marital status. For the total abortion ratio only, abortions for women of unknown marital status were distributed according to the distribution of abortions among women of known marital status.

### Previous Live Births and Previous Induced Abortions

Among the 43 areas that reported the number of previous live births for 2020, 39.1%, 24.5%, 20.3%, 9.7%, and 6.4% of abortions reported were among women who had zero, one, two, three, or four or more previous live births, respectively (Table 8). Among the 42 areas that reported the number of previous induced abortions for 2020, 57.7%, 24.1%, 10.5%, and 7.8% of abortions reported were among women who had had zero, one, two, or three or more previous induced abortions, respectively (Table 9).

**TABLE 8:** Number of reported abortions, by known number of previous live births and reporting area of occurrence — selected reporting areas,* United States, 2020

Area	No. of previous live births					Total abortions reported by known number of previous live births
	0	1	2	3	≥4	
	No. (%)^†^	No. (%)	No. (%)	No. (%)	No. (%)	No. (% of all reported abortions)^§^
Alabama	1,854 (32.5)	1,601 (28.0)	1,263 (22.1)	619 (10.8)	376 (6.6)	**5,713 (100.0)**
Alaska	559 (46.4)	241 (20.0)	194 (16.1)	133 (11.0)	79 (6.6)	**1,206 (100.0)**
Arizona	5,752 (43.7)	2,760 (21.0)	2,403 (18.3)	1,278 (9.7)	962 (7.3)	**13,155 (99.1)**
Arkansas	1,041 (33.0)	827 (26.2)	713 (22.6)	337 (10.7)	236 (7.5)	**3,154 (100.0)**
Colorado	5,549 (56.4)	1,846 (18.8)	1,395 (14.2)	654 (6.7)	386 (3.9)	**9,830 (99.6)**
Delaware	903 (39.6)	589 (25.8)	482 (21.1)	187 (8.2)	120 (5.3)	**2,281 (100.0)**
Florida	28,028 (37.4)	19,048 (25.4)	15,952 (21.3)	7,113 (9.5)	4,727 (6.3)	**74,868 (100.0)**
Georgia	14,273 (38.0)	9,300 (24.8)	7,644 (20.4)	3,717 (9.9)	2,594 (6.9)	**37,528 (100.0)**
Hawaii	980 (56.4)	286 (16.5)	264 (15.2)	126 (7.3)	81 (4.7)	**1,737 (96.0)**
Idaho	851 (50.7)	330 (19.7)	278 (16.6)	138 (8.2)	81 (4.8)	**1,678 (99.9)**
Indiana	2,890 (37.3)	1,947 (25.1)	1,622 (20.9)	809 (10.4)	487 (6.3)	**7,755 (100.0)**
Iowa	1,721 (42.4)	848 (20.9)	755 (18.6)	429 (10.6)	302 (7.4)	**4,055 (99.9)**
Kansas	3,005 (39.9)	1,762 (23.4)	1,475 (19.6)	797 (10.6)	487 (6.5)	**7,526 (100.0)**
Kentucky	1,379 (33.6)	1,091 (26.6)	932 (22.7)	426 (10.4)	276 (6.7)	**4,104 (100.0)**
Louisiana	2,227 (29.8)	2,025 (27.1)	1,739 (23.3)	908 (12.2)	574 (7.7)	**7,473 (100.0)**
Maine	997 (48.3)	443 (21.5)	383 (18.6)	160 (7.8)	81 (3.9)	**2,064 (100.0)**
Massachusetts	6,306 (43.3)	3,586 (24.6)	2,857 (19.6)	1,235 (8.5)	578 (4.0)	**14,562 (88.5)**
Michigan^¶^	9,856 (33.2)	7,724 (26.0)	6,631 (22.4)	3,224 (10.9)	2,220 (7.5)	**29,655 (100.0)**
Minnesota	3,986 (38.6)	2,435 (23.6)	2,080 (20.1)	1,067 (10.3)	764 (7.4)	**10,332 (99.8)**
Mississippi	1,048 (29.4)	1,028 (28.9)	776 (21.8)	434 (12.2)	273 (7.7)	**3,559 (100.0)**
Missouri	65 (38.9)	49 (29.3)	33 (19.8)	—**	—**	**167 (100.0)**
Montana	823 (49.2)	330 (19.7)	298 (17.8)	134 (8.0)	89 (5.3)	**1,674 (99.9)**
Nebraska	904 (38.0)	529 (22.3)	491 (20.7)	263 (11.1)	189 (8.0)	**2,376 (99.9)**
Nevada	3,672 (42.8)	1,841 (21.5)	1,661 (19.4)	800 (9.3)	604 (7.0)	**8,578 (99.4)**
New Jersey^††^	9,063 (39.5)	5,831 (25.4)	4,415 (19.2)	2,135 (9.3)	1,494 (6.5)	**22,938 (99.9)**
New Mexico	1,607 (41.5)	851 (22.0)	714 (18.5)	378 (9.8)	319 (8.2)	**3,869 (90.1)**
New York City	14,915 (43.5)	9,023 (26.3)	6,496 (18.9)	2,440 (7.1)	1,414 (4.1)	**34,288 (91.4)**
North Carolina	9,207 (34.6)	6,330 (23.8)	5,445 (20.5)	2,918 (11.0)	2,712 (10.2)	**26,612 (88.7)**
North Dakota	452 (38.5)	259 (22.1)	228 (19.4)	142 (12.1)	93 (7.9)	**1,174 (100.0)**
Ohio^§§^	6,485 (33.4)	5,010 (25.8)	4,326 (22.3)	2,140 (11.0)	1,447 (7.5)	**19,408 (94.2)**
Oklahoma	1,490 (39.3)	916 (24.2)	755 (19.9)	402 (10.6)	227 (6.0)	**3,790 (99.8)**
Oregon	3,608 (51.8)	1,377 (19.8)	1,149 (16.5)	506 (7.3)	326 (4.7)	**6,966 (99.6)**
Pennsylvania	11,674 (36.3)	8,349 (26.0)	6,726 (20.9)	3,306 (10.3)	2,068 (6.4)	**32,123 (100.0)**
Rhode Island	1,196 (45.8)	640 (24.5)	479 (18.4)	205 (7.9)	90 (3.4)	**2,610 (100.0)**
South Carolina	2,226 (40.7)	1,348 (24.7)	1,121 (20.5)	489 (8.9)	284 (5.2)	**5,468 (100.0)**
South Dakota	44 (35.2)	31 (24.8)	24 (19.2)	15 (12.0)	11 (8.8)	**125 (100.0)**
Texas	21,628 (39.2)	13,169 (23.9)	11,334 (20.6)	5,581 (10.1)	3,420 (6.2)	**55,132 (100.0)**
Utah	1,142 (48.4)	482 (20.4)	401 (17.0)	197 (8.3)	138 (5.8)	**2,360 (99.9)**
Vermont	630 (51.3)	252 (20.5)	207 (16.9)	91 (7.4)	47 (3.8)	**1,227 (100.0)**
Virginia	5,659 (36.3)	4,007 (25.7)	3,245 (20.8)	1,594 (10.2)	1,099 (7.0)	**15,604 (100.0)**
Washington	7,979 (47.3)	3,669 (21.7)	3,041 (18.0)	1,371 (8.1)	821 (4.9)	**16,881 (99.8)**
West Virginia	317 (31.7)	282 (28.2)	239 (23.9)	111 (11.1)	52 (5.2)	**1,001 (100.0)**
Wyoming	42 (46.7)	20 (22.2)	16 (17.8)	—**	—**	**90 (98.9)**
**Total**	**198,033 (39.1)**	**124,312 (24.5)**	**102,682 (20.3)**	**49,026 (9.7)**	**32,643 (6.4)**	**506,696 (98.0)** ^¶¶^

* Data from 43 reporting areas; excludes nine reporting areas (California, Connecticut, the District of Columbia, Illinois, Maryland, New Hampshire, New York State, Tennessee, and Wisconsin) that did not report, did not report by number of previous live births, or did not meet reporting standards.

^†^ Percentages for the individual component categories might not add to 100% because of rounding.

^§^ Percentage is calculated as the number of abortions reported by known number of previous live births divided by the sum of abortions reported by known and unknown number of previous live births. Values ≥99.95% are rounded to 100.0%.

^¶^ Recorded as the number of previous pregnancies carried to term.

** Cells with a numerical value in the range of 1–4 and cells that would allow for calculation of these small values have been suppressed.

^††^ Reporting to the central health agency is not required. Data are requested from hospitals and licensed ambulatory care facilities only.

^§§^ Recorded as the number of living children.

^¶¶^ Percentage based on a total of 517,261 abortions reported among the areas that met reporting standards for the number of previous live births.

**TABLE 9:** Number of reported abortions, by known number of previous induced abortions and reporting area of occurrence — selected reporting areas,* United States, 2020

Area	No. of previous induced abortions				Total abortions reported by known number of previous induced abortions
	0	1	2	≥3	
	No. (%)^†^	No. (%)	No. (%)	No. (%)	No. (% of all reported abortions)^§^
Alabama	3,721 (65.1)	1,300 (22.8)	465 (8.1)	227 (4.0)	**5,713 (100.0)**
Alaska	788 (65.3)	277 (23.0)	83 (6.9)	58 (4.8)	**1,206 (100.0)**
Arizona	8,557 (65.2)	3,108 (23.7)	1,013 (7.7)	442 (3.4)	**13,120 (98.8)**
Arkansas	2,011 (63.8)	635 (20.1)	293 (9.3)	215 (6.8)	**3,154 (100.0)**
Colorado	6,784 (68.8)	2,111 (21.4)	642 (6.5)	328 (3.3)	**9,865 (100.0)**
Delaware	1,391 (61.0)	529 (23.2)	215 (9.4)	145 (6.4)	**2,280 (100.0)**
Florida	41,715 (55.7)	18,992 (25.4)	8,142 (10.9)	6,019 (8.0)	**74,868 (100.0)**
Georgia	23,266 (62.0)	8,693 (23.2)	3,522 (9.4)	2,047 (5.5)	**37,528 (100.0)**
Hawaii	1,128 (64.9)	379 (21.8)	138 (7.9)	92 (5.3)	**1,737 (96.0)**
Idaho	1,339 (79.7)	250 (14.9)	66 (3.9)	24 (1.4)	**1,679 (99.9)**
Indiana	5,292 (68.2)	1,628 (21.0)	572 (7.4)	263 (3.4)	**7,755 (100.0)**
Iowa	2,771 (68.3)	798 (19.7)	298 (7.3)	188 (4.6)	**4,055 (99.9)**
Kansas	5,265 (70.0)	1,473 (19.6)	507 (6.7)	281 (3.7)	**7,526 (100.0)**
Kentucky	2,685 (65.4)	925 (22.5)	310 (7.6)	184 (4.5)	**4,104 (100.0)**
Louisiana	4,589 (61.4)	1,900 (25.4)	640 (8.6)	344 (4.6)	**7,473 (100.0)**
Maine	1,364 (66.2)	459 (22.3)	161 (7.8)	77 (3.7)	**2,061 (99.9)**
Massachusetts	8,263 (52.1)	4,264 (26.9)	1,931 (12.2)	1,391 (8.8)	**15,849 (96.3)**
Michigan	15,291 (51.6)	7,421 (25.0)	3,975 (13.4)	2,969 (10.0)	**29,656 (100.0)**
Minnesota	5,988 (58.0)	2,485 (24.0)	1,056 (10.2)	804 (7.8)	**10,333 (99.8)**
Mississippi	2,388 (67.1)	806 (22.6)	269 (7.6)	96 (2.7)	**3,559 (100.0)**
Missouri	127 (77.4)	23 (14.0)	9 (5.5)	5 (3.0)	**164 (98.2)**
Montana	722 (43.1)	631 (37.7)	218 (13.0)	104 (6.2)	**1,675 (100.0)**
Nebraska	1,630 (68.5)	496 (20.9)	148 (6.2)	104 (4.4)	**2,378 (100.0)**
Nevada	5,070 (59.2)	1,939 (22.7)	898 (10.5)	650 (7.6)	**8,557 (99.1)**
New Jersey^¶^	12,844 (55.9)	4,995 (21.7)	2,572 (11.2)	2,559 (11.1)	**22,970 (100.0)**
New York City	12,826 (38.2)	8,133 (24.2)	5,757 (17.1)	6,897 (20.5)	**33,613 (89.6)**
North Carolina	15,089 (56.8)	6,900 (26.0)	2,912 (11.0)	1,651 (6.2)	**26,552 (88.5)**
North Dakota	778 (66.3)	255 (21.7)	93 (7.9)	48 (4.1)	**1,174 (100.0)**
Ohio	11,085 (57.7)	4,757 (24.8)	2,056 (10.7)	1,304 (6.8)	**19,202 (93.2)**
Oklahoma	2,631 (69.5)	784 (20.7)	247 (6.5)	126 (3.3)	**3,788 (99.8)**
Oregon	4,408 (63.3)	1,535 (22.1)	598 (8.6)	418 (6.0)	**6,959 (99.5)**
Pennsylvania	16,900 (52.6)	7,839 (24.4)	3,917 (12.2)	3,467 (10.8)	**32,123 (100.0)**
Rhode Island	1,513 (58.0)	640 (24.5)	278 (10.7)	178 (6.8)	**2,609 (99.9)**
South Carolina	3,629 (66.4)	1,143 (20.9)	446 (8.2)	250 (4.6)	**5,468 (100.0)**
South Dakota	82 (65.6)	28 (22.4)	10 (8.0)	5 (4.0)	**125 (100.0)**
Texas	34,757 (63.0)	13,278 (24.1)	4,669 (8.5)	2,428 (4.4)	**55,132 (100.0)**
Utah	1,742 (74.3)	477 (20.3)	101 (4.3)	25 (1.1)	**2,345 (99.3)**
Vermont	817 (66.6)	267 (21.8)	91 (7.4)	52 (4.2)	**1,227 (100.0)**
Virginia	8,087 (51.8)	4,585 (29.4)	1,776 (11.4)	1,156 (7.4)	**15,604 (100.0)**
Washington	10,056 (59.5)	3,910 (23.1)	1,612 (9.5)	1,322 (7.8)	**16,900 (99.9)**
West Virginia	636 (63.5)	232 (23.2)	77 (7.7)	56 (5.6)	**1,001 (100.0)**
Wyoming	65 (71.4)	21 (23.1)	5 (5.5)	0 (0.0)	**91 (100.0)**
**Total**	**290,090 (57.7)**	**121,301 (24.1)**	**52,788 (10.5)**	**38,999 (7.8)**	503,178 (98.1)**

* Data from 42 reporting areas; excludes 10 reporting areas (California, Connecticut, the District of Columbia, Illinois, Maryland, New Hampshire, New Mexico, New York State, Tennessee, and Wisconsin) that did not report, did not report by number of previous induced abortions, or did not meet reporting standards.

^†^ Percentages for the individual component categories might not add to 100% because of rounding.

^§^ Percentage is calculated as the number of abortions reported by known number of previous induced abortions divided by the sum of abortions reported by known and unknown number of previous induced abortions. Values ≥99.95% are rounded to 100.0%.

^¶^ Reporting to the central health agency is not required. Data are requested from hospitals and licensed ambulatory care facilities only.

** Percentage based on a total of 512,968 abortions reported among the areas that met reporting standards for the number of previous induced abortions.

### Weeks of Gestation and Method Type

Among the 41 areas that reported gestational age at the time of abortion for 2020, 80.9% of abortions were performed at ≤9 weeks’ gestation, and nearly all (93.1%) were performed at ≤13 weeks’ gestation (Table 10). Fewer abortions were performed at 14–20 weeks’ gestation (5.8%) or at ≥21 weeks’ gestation (0.9%). Among the 33 reporting areas that provided data every year on gestational age for 2011–2020, the percentage of abortions performed at ≤13 weeks’ gestation changed from 91.3% to 92.5% (Table 11). However, within this gestational age range, a shift occurred toward earlier gestational ages, with the percentage of abortions performed at ≤6 weeks’ gestation increasing 17% and the percentage of abortions performed at 7–9 weeks’ and 10–13 weeks’ gestation decreasing 2% and 21%, respectively.

**TABLE 10:** Number of reported abortions, by known weeks of gestation* and reporting area of occurrence — selected reporting areas,^†^ United States, 2020

Area	Weeks of gestation							Total abortions reported by known gestational age
	≤6	7–9	10–13	14–15	16–17	18–20	≥21	
	No. (%)^§^	No. (%)	No. (%)	No. (%)	No. (%)	No. (%)	No. (%)	No. (% of all reported abortions)^¶^
Alabama**	1,333 (23.4)	2,746 (48.2)	1,072 (18.8)	234 (4.1)	144 (2.5)	130 (2.3)	37 (0.6)	**5,696 (99.7)**
Alaska	308 (25.5)	614 (50.9)	198 (16.4)	56 (4.6)	30 (2.5)	0 (—)	0 (—)	**1,206 (100.0)**
Arizona	3,799 (28.6)	6,252 (47.1)	2,060 (15.5)	524 (3.9)	211 (1.6)	258 (1.9)	169 (1.3)	**13,273 (100.0)**
Arkansas**	429 (13.6)	1,471 (46.6)	915 (29.0)	138 (4.4)	90 (2.9)	85 (2.7)	26 (0.8)	**3,154 (100.0)**
Colorado	4,671 (47.3)	3,495 (35.4)	970 (9.8)	195 (2.0)	147 (1.5)	130 (1.3)	261 (2.6)	**9,869 (100.0)**
Delaware	628 (27.6)	1,209 (53.2)	337 (14.8)	79 (3.5)	9 (0.4)	—^††^	—^††^	**2,272 (99.6)**
Florida	55,834 (74.6)	11,686 (15.6)	4,768 (6.4)	1,005 (1.3)	652 (0.9)	704 (0.9)	219 (0.3)	**74,868 (100.0)**
Georgia	17,478 (46.6)	14,184 (37.8)	3,877 (10.3)	833 (2.2)	483 (1.3)	526 (1.4)	152 (0.4)	**37,533 (100.0)**
Hawaii	717 (39.6)	719 (39.7)	215 (11.9)	50 (2.8)	42 (2.3)	36 (2.0)	30 (1.7)	**1,809 (100.0)**
Idaho	512 (30.6)	814 (48.6)	271 (16.2)	53 (3.2)	16 (1.0)	—^††^	—^††^	**1,675 (99.7)**
Indiana	2,061 (26.6)	4,244 (54.7)	1,382 (17.8)	14 (0.2)	10 (0.1)	19 (0.2)	26 (0.3)	**7,756 (100.0)**
Iowa	2,001 (49.3)	1,407 (34.7)	442 (10.9)	79 (1.9)	67 (1.7)	51 (1.3)	11 (0.3)	**4,058 (100.0)**
Kansas	3,120 (41.5)	2,748 (36.5)	1,092 (14.5)	238 (3.2)	135 (1.8)	152 (2.0)	40 (0.5)	**7,525 (100.0)**
Kentucky	1,320 (32.2)	1,773 (43.2)	590 (14.4)	147 (3.6)	104 (2.5)	135 (3.3)	35 (0.9)	**4,104 (100.0)**
Louisiana	2,351 (31.5)	3,185 (42.6)	1,389 (18.6)	316 (4.2)	179 (2.4)	52 (0.7)	0 (—)	**7,472 (100.0)**
Maine	746 (36.2)	955 (46.3)	267 (12.9)	43 (2.1)	27 (1.3)	25 (1.2)	0 (—)	**2,063 (100.0)**
Michigan	11,427 (38.7)	11,775 (39.8)	3,891 (13.2)	956 (3.2)	643 (2.2)	524 (1.8)	349 (1.2)	**29,565 (99.6)**
Minnesota	4,202 (41.4)	3,813 (37.5)	1,213 (11.9)	347 (3.4)	201 (2.0)	186 (1.8)	200 (2.0)	**10,162 (98.2)**
Mississippi	1,181 (33.2)	1,716 (48.2)	485 (13.6)	160 (4.5)	14 (0.4)	—^††^	—^††^	**3,559 (100.0)**
Missouri	6 (3.6)	30 (18.0)	—^††^	22 (13.2)	—^††^	24 (14.4)	39 (23.4)	**167 (100.0)**
Montana	717 (43.0)	658 (39.4)	201 (12.0)	39 (2.3)	29 (1.7)	—^††^	—^††^	**1,669 (99.6)**
Nebraska	963 (40.5)	914 (38.4)	330 (13.9)	86 (3.6)	43 (1.8)	30 (1.3)	12 (0.5)	**2,378 (100.0)**
Nevada	3,426 (40.0)	3,405 (39.7)	1,119 (13.1)	239 (2.8)	150 (1.7)	138 (1.6)	97 (1.1)	**8,574 (99.3)**
New Jersey^§§^	9,704 (43.2)	7,927 (35.3)	2,567 (11.4)	840 (3.7)	550 (2.4)	461 (2.1)	424 (1.9)	**22,473 (97.8)**
New Mexico	1,467 (40.0)	1,081 (29.5)	419 (11.4)	103 (2.8)	90 (2.5)	132 (3.6)	372 (10.2)	**3,664 (85.3)**
New York City	16,732 (44.6)	13,281 (35.4)	4,256 (11.3)	1,003 (2.7)	652 (1.7)	825 (2.2)	773 (2.1)	**37,522 (100.0)**
North Carolina	11,310 (38.2)	12,314 (41.6)	4,118 (13.9)	870 (2.9)	503 (1.7)	473 (1.6)	48 (0.2)	**29,636 (98.8)**
North Dakota	448 (38.2)	483 (41.1)	183 (15.6)	47 (4.0)	11 (0.9)	—^††^	—^††^	**1,174 (100.0)**
Ohio	5,695 (27.6)	9,396 (45.6)	3,405 (16.5)	891 (4.3)	562 (2.7)	543 (2.6)	113 (0.5)	**20,605 (100.0)**
Oklahoma	1,768 (46.9)	1,362 (36.1)	484 (12.8)	69 (1.8)	28 (0.7)	48 (1.3)	14 (0.4)	**3,773 (99.4)**
Oregon	3,286 (47.0)	2,436 (34.9)	761 (10.9)	181 (2.6)	120 (1.7)	95 (1.4)	110 (1.6)	**6,989 (100.0)**
Rhode Island	983 (37.7)	1,065 (40.9)	373 (14.3)	66 (2.5)	55 (2.1)	48 (1.8)	16 (0.6)	**2,606 (99.8)**
South Carolina**	1,210 (22.1)	2,003 (36.6)	1,936 (35.4)	294 (5.4)	—^††^	—^††^	12 (0.2)	**5,468 (100.0)**
South Dakota	17 (13.7)	55 (44.4)	49 (39.5)	—^††^	—^††^	—^††^	—^††^	**124 (99.2)**
Texas**	22,093 (40.1)	21,849 (39.6)	7,205 (13.1)	1,902 (3.4)	1,016 (1.8)	784 (1.4)	283 (0.5)	**55,132 (100.0)**
Utah	857 (36.3)	864 (36.6)	430 (18.2)	83 (3.5)	48 (2.0)	51 (2.2)	29 (1.2)	**2,362 (100.0)**
Vermont	590 (48.1)	455 (37.1)	97 (7.9)	37 (3.0)	13 (1.1)	18 (1.5)	16 (1.3)	**1,226 (99.9)**
Virginia	8,578 (55.0)	4,883 (31.3)	1,695 (10.9)	101 (0.6)	99 (0.6)	139 (0.9)	108 (0.7)	**15,603 (100.0)**
Washington	7,291 (43.2)	6,457 (38.3)	1,818 (10.8)	433 (2.6)	247 (1.5)	279 (1.7)	346 (2.1)	**16,871 (99.8)**
West Virginia	286 (28.6)	440 (44.0)	195 (19.5)	57 (5.7)	15 (1.5)	—^††^	—^††^	**1,001 (100.0)**
Wyoming	49 (54.4)	38 (42.2)	—^††^	—^††^	0 (—)	0 (—)	0 (—)	**90 (98.9)**
**Total**	**211,594 (45.3)**	**166,202 (35.6)**	**57,108 (12.2)**	**12,830 (2.7)**	**7,456 (1.6)**	**7,154 (1.5)**	**4,382 (0.9)**	**466,726 (99.6)** ^¶¶^

* Gestational age based on clinician’s estimate (Alaska, Arizona, Colorado, Delaware, Florida, Georgia, Hawaii, Idaho, Indiana, Iowa, Kansas, Kentucky, Louisiana, Maine, Michigan, Minnesota, Mississippi, Missouri, Montana, Nebraska, Nevada, New Jersey, New Mexico, New York City, North Carolina, North Dakota, Ohio, Oregon, Rhode Island, South Dakota, Vermont, Washington, West Virginia, and Wyoming); gestational age calculated from the last normal menstrual period (Oklahoma and Utah); clinician’s estimate of gestation based on estimated date of conception (Virginia); and probable postfertilization age (Alabama, Arkansas, South Carolina, and Texas).

^†^ Data from 41 reporting areas; excludes 11 reporting areas (California, Connecticut, the District of Columbia, Illinois, Maryland, Massachusetts, New Hampshire, New York State, Pennsylvania, Tennessee, and Wisconsin) that did not report, did not report by gestational age, or did not meet reporting standards.

^§^ Percentages for the individual component categories might not add to 100% because of rounding.

^¶^ Percentage is calculated as the number of abortions reported by known gestational age divided by the sum of abortions reported by known and unknown gestational age. Values ≥99.95% are rounded to 100.0%.

** Two weeks were added to the probable postfertilization age to provide a corresponding measure to gestational age based on the clinician’s estimate.

^††^ Cells with a numerical value in the range of 1–4 and cells that would allow for calculation of these small values have been suppressed.

^§§^ Reporting to the central health agency is not required. Data are requested from hospitals and licensed ambulatory care facilities only.

^¶¶^ Percentage based on a total of 468,686 abortions reported among the areas that met reporting standards for gestational age.

**TABLE 11:** Percentage of reported abortions, by known weeks of gestation and year — selected reporting areas,* United States, 2011–2020

Weeks of gestation	Year										% Change	
	2011	2012	2013	2014	2015	2016	2017	2018	2019	2020	2019 to 2020	2011 to 2020
**≤13 weeks’ gestation (%)^†^**	**91.3**	**91.2**	**91.4**	**90.8**	**90.9**	**90.9**	**91.1**	**91.5**	**92.0**	**92.5**	**0.5**	**1.3**
≤6	34.2	34.9	34.6	33.8	34.4	34.6	35.5	36.8	38.0	39.9	5.0	16.7
7–9	40.1	39.5	39.9	39.9	39.9	40.1	40.1	39.7	39.6	39.2	−1.0	−2.2
10–13	17.0	16.9	16.9	17.1	16.5	16.2	15.5	15.0	14.4	13.4	−6.9	−21.2
**>13 weeks’ gestation (%)^†^**	**8.7**	**8.8**	**8.6**	**9.2**	**9.1**	**9.1**	**8.9**	**8.5**	**8.0**	**7.5**	**−6.3**	**−13.8**
14–15	3.4	3.5	3.4	3.6	3.5	3.5	3.4	3.3	3.2	3.0	−6.3	−11.8
16–17	1.9	1.9	2.0	2.3	2.2	2.2	2.2	2.0	1.9	1.8	−5.3	−5.3
18–20	1.9	2.0	1.9	2.0	2.1	2.1	2.0	1.9	1.8	1.7	−5.6	−10.5
≥21	1.4	1.4	1.4	1.4	1.4	1.3	1.3	1.2	1.2	1.1	−8.3	−21.4
**Total no.^§^**	**465,754**	**441,667**	**421,900**	**414,437**	**403,641**	**397,773**	**383,417**	**385,163**	**388,802**	**377,664**	**N/A**	**N/A**

**Abbreviation:** N/A = not applicable.

* Data from 33 reporting areas; excludes 19 areas (California, Connecticut, Delaware, the District of Columbia, Florida, Illinois, Maine, Maryland, Massachusetts, Mississippi, Nebraska, New Hampshire, New York State, Pennsylvania, Rhode Island, Tennessee, Vermont, Wisconsin, and Wyoming) that did not report, did not report by weeks of gestation, or did not meet reporting standards for ≥1 year.

^†^ Percentages for the individual component categories might not add to 100% because of rounding.

^§^ By year, the total number of abortions represent 72.8%–94.4% of all abortions reported to CDC among the areas that met reporting standards for gestational age during 2011–2020.

Among the 46 areas that reported by method type for 2020 and included medical abortion on their reporting form, 51.0% were early medical abortions (a nonsurgical abortion at ≤9 weeks’ gestation), 40.0% of abortions were surgical abortions at ≤13 weeks’ gestation, 6.7% were surgical abortions at >13 weeks’ gestation, and 2.4% were medical abortions at >9 weeks’ gestation; other methods, including intrauterine instillation and hysterectomy/hysterotomy, were rare (<0.1%) (Table 12). During 2011−2020, a total of 37 reporting areas (excludes Alabama, California, the District of Columbia, Florida, Hawaii, Illinois, Louisiana, Maine, Maryland, New Hampshire, New Mexico, Tennessee, Vermont, Wisconsin, and Wyoming) provided continuous data and included medical abortion on their reporting form. Among these 37 areas, use of early medical abortion increased 22% from 2019 to 2020 (from 41.1% to 50.0% of abortions) and 154% from 2011 to 2020 (from 19.7% to 50.0% of abortions).

**TABLE 12:** Number of reported abortions, by known method type and reporting area of occurrence — selected reporting areas,* United States, 2020

Area	Surgical^†^			Medical			Intrauterine instillation^§^	Hysterectomy/ hysterotomy	Total abortions reported by known method type
	Surgical, ≤13 weeks’ gestation	Surgical, >13 weeks’ gestation	Surgical, unknown gestational age	Medical, ≤9 weeks’ gestation	Medical, >9 weeks’ gestation	Medical, unknown gestational age			
	No. (%)^¶^	No. (%)	No. (%)	No. (%)	No. (%)	No. (%)	No. (%)	No. (%)	No. (% of all reported abortions)**
Alabama^††^	2,641 (46.3)	541 (9.5)	8 (0.1)	2,411 (42.3)	94 (1.6)	9 (0.2)	0 (—)	0 (—)	**5,704 (99.8)**
Alaska	669 (55.5)	85 (7.1)	—**^§§^**	433 (35.9)	17 (1.4)	0 (—)	0 (—)	—**^§§^**	**1,205 (99.9)**
Arizona	5,506 (41.5)	933 (7.0)	0 (—)	6,329 (47.7)	343 (2.6)	0 (—)	154 (1.2)	0 (—)	**13,265 (99.9)**
Arkansas^††^	1,091 (34.6)	338 (10.7)	0 (—)	1,236 (39.2)	489 (15.5)	0 (—)	0 (—)	0 (—)	**3,154 (100.0)**
Colorado	2,320 (25.2)	390 (4.2)	0 (—)	6,217 (67.6)	263 (2.9)	0 (—)	0 (—)	0 (—)	**9,190 (93.1)**
Connecticut^¶¶^	N/A	N/A	3,157 (34.7)	N/A	N/A	5,942 (65.3)	—**^§§^**	—**^§§^**	**9,100 (99.8)**
Delaware	672 (29.7)	94 (4.2)	—**^§§^**	1,385 (61.3)	100 (4.4)	7 (0.3)	—**^§§^**	0 (—)	**2,260 (99.1)**
District of Columbia***	1,737 (39.3)	321 (7.3)	0 (—)	N/A	N/A	2,358 (53.4)	0 (—)	0 (—)	**4,416 (100.0)**
Florida	27,408 (38.2)	2,489 (3.5)	0 (—)	41,395 (57.7)	436 (0.6)	0 (—)	0 (—)	6 (0.0)	**71,734 (95.8)**
Georgia	12,673 (33.8)	1,984 (5.3)	0 (—)	22,174 (59.1)	702 (1.9)	0 (—)	0 (—)	0 (—)	**37,533 (100.0)**
Hawaii	767 (42.4)	158 (8.7)	0 (—)	875 (48.4)	9 (0.5)	0 (—)	0 (—)	0 (—)	**1,809 (100.0)**
Idaho	708 (42.2)	75 (4.5)	—**^§§^**	841 (50.1)	47 (2.8)	—**^§§^**	—**^§§^**	0 (—)	**1,677 (99.8)**
Indiana	3,439 (44.3)	58 (0.7)	0 (—)	4,165 (53.7)	94 (1.2)	0 (—)	0 (—)	0 (—)	**7,756 (100.0)**
Iowa	634 (15.6)	201 (5.0)	0 (—)	3,071 (75.7)	151 (3.7)	0 (—)	0 (—)	0 (—)	**4,057 (100.0)**
Kansas	1,918 (25.5)	561 (7.5)	—**^§§^**	4,749 (63.1)	297 (3.9)	—**^§§^**	0 (—)	0 (—)	**7,526 (100.0)**
Kentucky	1,598 (38.9)	419 (10.2)	0 (—)	2,080 (50.7)	7 (0.2)	0 (—)	0 (—)	0 (—)	**4,104 (100.0)**
Maine	527 (25.5)	92 (4.5)	—**^§§^**	1,343 (65.1)	101 (4.9)	—**^§§^**	0 (—)	0 (—)	**2,064 (100.0)**
Massachusetts^†††^	N/A	N/A	8,669 (52.7)	N/A	N/A	7,773 (47.3)	—**^§§^**	—**^§§^**	**16,443 (99.9)**
Michigan	11,771 (39.8)	2,377 (8.0)	54 (0.2)	14,444 (48.9)	844 (2.9)	45 (0.2)	—**^§§^**	—**^§§^**	**29,539 (99.6)**
Minnesota	3,688 (35.7)	913 (8.8)	50 (0.5)	5,221 (50.5)	335 (3.2)	135 (1.3)	0 (—)	0 (—)	**10,342 (99.9)**
Mississippi	388 (10.9)	170 (4.8)	—**^§§^**	2,788 (78.3)	212 (6.0)	0 (—)	0 (—)	—**^§§^**	**3,559 (100.0)**
Missouri	—**^§§^**	70 (43.5)	0 (—)	0 (—)	22 (13.7)	0 (—)	—**^§§^**	—**^§§^**	**161 (96.4)**
Montana	488 (29.1)	91 (5.4)	—**^§§^**	1,037 (61.9)	51 (3.0)	6 (0.4)	0 (—)	—**^§§^**	**1,675 (100.0)**
Nebraska	527 (22.2)	167 (7.0)	0 (—)	1,602 (67.4)	81 (3.4)	0 (—)	0 (—)	0 (—)	**2,377 (100.0)**
Nevada	3,357 (40.0)	617 (7.4)	27 (0.3)	4,133 (49.3)	219 (2.6)	27 (0.3)	—**^§§^**	—**^§§^**	**8,383 (97.1)**
New Jersey^§§§^	11,156 (48.6)	2,238 (9.7)	340 (1.5)	8,783 (38.2)	296 (1.3)	159 (0.7)	0 (—)	0 (—)	**22,972 (100.0)**
New Mexico	1,171 (31.4)	364 (9.8)	117 (3.1)	1,579 (42.3)	351 (9.4)	149 (4.0)	0 (—)	0 (—)	**3,731 (86.9)**
New York	30,161 (49.0)	4,948 (8.0)	1,234 (2.0)	20,588 (33.4)	2,229 (3.6)	2,287 (3.7)	72 (0.1)	34 (0.1)	**61,553 (97.5)**
New York City	20,957 (55.9)	3,094 (8.2)	—**^§§^**	12,850 (34.2)	558 (1.5)	—**^§§^**	25 (0.1)	34 (0.1)	**37,519 (100.0)**
New York State	9,204 (38.3)	1,854 (7.7)	—**^§§^**	7,738 (32.2)	1,671 (7.0)	—**^§§^**	47 (0.2)	0 (—)	**24,034 (93.8)**
North Carolina	9,731 (33.8)	1,818 (6.3)	62 (0.2)	16,395 (56.9)	735 (2.6)	72 (0.2)	—**^§§^**	—**^§§^**	**28,822 (96.1)**
North Dakota	372 (31.7)	58 (4.9)	—**^§§^**	740 (63.0)	—**^§§^**	0 (—)	0 (—)	0 (—)	**1,174 (100.0)**
Ohio	8,659 (42.0)	2,090 (10.1)	—**^§§^**	9,711 (47.1)	140 (0.7)	0 (—)	0 (—)	—**^§§^**	**20,602 (100.0)**
Oklahoma	1,087 (29.3)	154 (4.2)	5 (0.1)	2,287 (61.7)	156 (4.2)	19 (0.5)	0 (—)	0 (—)	**3,708 (97.7)**
Oregon	2,407 (34.6)	447 (6.4)	—**^§§^**	3,911 (56.1)	197 (2.8)	—**^§§^**	0 (—)	—**^§§^**	**6,966 (99.6)**
Pennsylvania^¶¶^	N/A	N/A	15,757 (49.1)	N/A	N/A	16,349 (50.9)	—**^§§^**	—**^§§^**	**32,115 (100.0)**
Rhode Island	1,084 (41.6)	182 (7.0)	—**^§§^**	1,252 (48.0)	84 (3.2)	—**^§§^**	—**^§§^**	0 (—)	**2,608 (99.9)**
South Carolina^††^	1,463 (26.8)	311 (5.7)	0 (—)	2,546 (46.6)	1,142 (20.9)	0 (—)	—**^§§^**	—**^§§^**	**5,467 (100.0)**
South Dakota	75 (60.5)	—**^§§^**	0 (—)	41 (33.1)	7 (5.6)	—**^§§^**	0 (—)	0 (—)	**124 (99.2)**
Texas^††^	22,331 (40.5)	3,932 (7.1)	—**^§§^**	28,359 (51.4)	507 (0.9)	0 (—)	0 (—)	—**^§§^**	**55,130 (100.0)**
Utah	1,252 (53.0)	202 (8.6)	0 (—)	878 (37.2)	30 (1.3)	0 (—)	0 (—)	0 (—)	**2,362 (100.0)**
Vermont	223 (18.2)	77 (6.3)	—**^§§^**	890 (72.6)	35 (2.9)	—**^§§^**	0 (—)	0 (—)	**1,226 (99.9)**
Virginia	7,673 (49.3)	441 (2.8)	—**^§§^**	7,315 (47.0)	147 (0.9)	—**^§§^**	0 (—)	0 (—)	**15,577 (99.8)**
Washington	6,305 (37.4)	1,288 (7.6)	12 (0.1)	9,023 (53.5)	219 (1.3)	25 (0.1)	0 (—)	0 (—)	**16,872 (99.8)**
West Virginia	421 (42.1)	66 (6.6)	0 (—)	445 (44.5)	69 (6.9)	0 (—)	0 (—)	0 (—)	**1,001 (100.0)**
Wisconsin^¶¶,¶¶¶^	N/A	N/A	3,837 (60.6)	N/A	N/A	2,499 (39.4)	0 (—)	0 (—)	**6,336 (100.0)**
Wyoming	—**^§§^**	0 (—)	0 (—)	86 (96.6)	—**^§§^**	0 (—)	0 (—)	0 (—)	**89 (97.8)**
**Total**	**218,734 (40.0)**	**36,531 (6.7)**	—****	**278,947 (51.0)**	**12,943 (2.4)**	**—^††††^**	**241 (0.0)**	**72 (0.0)**	**547,468 (98.6)** ^§§§§^

**Abbreviation:** N/A = not applicable.

* Data from 46 reporting areas; excludes six reporting areas (California, Illinois, Louisiana, Maryland, New Hampshire, and Tennessee) that did not report, did not report by method type, or did not meet reporting standards. Areas reporting by method type with unknown gestational age or gestational age reported was not compatible with categorizations presented in this table are not included.

**^†^** Includes uterine aspiration (might also be called dilation and curettage, aspiration curettage, suction curettage, manual vacuum aspiration, menstrual extraction, sharp curettage) and dilation and evacuation procedures.

^§^ Intrauterine instillations reported at ≤12 weeks’ gestation were considered as unknown for method type.

^¶^ Percentages for the individual component categories might not add to 100% because of rounding.

** Percentage is calculated as the number of abortions reported by known method type divided by the sum of abortions reported by known and unknown method type. Values ≥99.95% are rounded to 100.0%.

^††^ Two weeks were added to the probable postfertilization age to provide a corresponding measure to gestational age based on the clinician’s estimate.

^§§^ Cells with a numerical value in the range of 1–4 and cells that would allow for calculation of these small values have been suppressed.

^¶¶^ Numbers for surgical abortions ≤13 weeks and >13 weeks and medical abortions ≤9 weeks versus >9 weeks are not presented as gestational age reported was not compatible with these categorizations.

*** Gestational age based on clinician’s estimate. Numbers for medical abortions at ≤9 weeks versus >9 weeks are not presented because gestational age reported was not compatible with these categorizations.

^†††^ Numbers for surgical abortions at ≤13 weeks versus >13 weeks and for medical abortions at ≤9 weeks versus >9 weeks are not presented because gestational age data were not provided by method type.

^§§§^ Reporting to the central health agency is not required. Data are requested from hospitals and licensed ambulatory care facilities only.

^¶¶¶^ Includes residents only. Wisconsin reports as surgical, unspecified and does not differentiate surgical abortions from hysterectomy/hysterotomy. All abortions were reported as surgical or chemically induced. For this report, all surgical abortions were classified as surgical and all chemical abortions as medical.

**** For the total only, surgical abortions reported without a gestational age were distributed among the surgical abortion categories according to the distribution of surgical abortions at known gestational age.

^††††^ For the total only, medical abortions reported without a gestational age were distributed among the medical abortion categories according to the distribution of medical abortions at known gestational age.

^§§§§^ Percentage based on a total of 555,274 abortions reported among the areas that met reporting standards for method type.

Among the 40 areas that reported abortions categorized by individual weeks of gestation and method type for 2020, surgical abortion accounted for the highest percentage of abortions at >10 weeks’ gestation (Table 13). Surgical abortion accounted for 32.1% of abortions at ≤6 weeks’ gestation, 41.2% of abortions at 7–9 weeks’ gestation, 84.3% of abortions at 10–13 weeks’ gestation, 96.2%–98.8% of abortions at 14–20 weeks’ gestation, and 86.3% of abortions at ≥21 weeks’ gestation. In contrast, medical abortion accounted for 67.9% of abortions at ≤6 weeks’ gestation, 58.7% of abortions at 7–9 weeks’ gestation, 15.7% of abortions at 10–13 weeks’ gestation, 1.2%–2.9% of abortions at 14–20 weeks’ gestation, and 11.8% of abortions at ≥21 weeks’ gestation. For each gestational age category as applicable, abortions performed by intrauterine instillation or hysterectomy/hysterotomy were rare (<0.1%–1.7% of abortions).

**TABLE 13:** Number of reported abortions, by known weeks of gestation and method type — selected reporting areas,* United States, 2020

Method type	Weeks of gestation							Total
	≤6	7–9	10–13	14–15	16–17	18–20	≥21	
	No. (%)^†^	No. (%)	No. (%)	No. (%)	No. (%)	No. (%)	No. (%)	No. (%)
**Surgical^§^**								
≤13 weeks’ gestation	66,200 (32.1)	66,659 (41.2)	46,364 (84.3)	N/A	N/A	N/A	N/A	**179,223 (39.5)**
>13 weeks’ gestation	N/A	N/A	N/A	12,282 (98.8)	7,068 (97.7)	6,724 (96.2)	3,511 (86.3)	**29,585 (6.5)**
**Medical^¶^**								
≤9 weeks’ gestation	140,098 (67.9)	94,922 (58.7)	N/A	N/A	N/A	N/A	N/A	**235,020 (51.8)**
>9 weeks’ gestation	N/A	N/A	8,645 (15.7)	150 (1.2)	116 (1.6)	200 (2.9)	482 (11.8)	**9,593 (2.1)**
**Intrauterine instillation**	—**	—**	5 (0.0)	1 (0.0)	50 (0.7)	63 (0.9)	70 (1.7)	**189 (0.0)**
**Hysterectomy/Hysterotomy**	17 (0.0)	26 (0.0)	9 (0.0)	1 (0.0)	1 (0.0)	5 (0.1)	7 (0.2)	**66 (0.0)**
**Total**	**206,315 (100.0)**	**161,607 (100.0)**	**55,023 (100.0)**	**12,434 (100.0)**	**7,235 (100.0)**	**6,992 (100.0)**	**4,070 (100.0)**	**453,676 (100.0)**

**Abbreviation:** N/A = not applicable.

* Data from 40 reporting areas; excludes 12 reporting areas (California, Connecticut, the District of Columbia, Illinois, Louisiana, Maryland, Massachusetts, New Hampshire, New York State, Pennsylvania, Tennessee, and Wisconsin) that did not report, did not report by weeks of gestation, did not meet reporting standards, or did not have medical abortion as a specific category on their reporting form.

^†^ For each gestational age category, percentages of all method types might not add to 100% because of rounding.

^§^ Includes uterine aspiration (might also be called dilation and curettage, aspiration curettage, suction curettage, manual vacuum aspiration, menstrual extraction, sharp curettage) and dilation and evacuation procedures.

^¶^ The administration of medication or medications to induce an abortion; at ≤9 weeks’ gestation, typically involves the use of mifepristone and misoprostol and at >9 weeks’ gestation, typically involves the use of vaginal prostaglandins.

** Intrauterine instillations reported at ≤12 weeks’ gestation have not been included with known values.

### Weeks of Gestation by Age Group and Race or Ethnicity

In reporting areas that provided data that met CDC reporting standards, abortions that were categorized by weeks of gestation were further categorized by age and by race or ethnicity (Table 14). In every subgroup for these characteristics, the highest percentage of abortions occurred at ≤9 weeks’ gestation. In 41 reporting areas, by age, 61.0% of adolescents aged <15 years and 75.5% of adolescents aged 15–19 years obtained an abortion at ≤9 weeks’ gestation, compared with ≥80.6% among women aged ≥20 years. Conversely, 18.7% of adolescents aged <15 years and 9.1% of adolescents aged 15–19 years obtained an abortion after 13 weeks’ gestation, compared with 6.3%–7.2% for women aged ≥20 years. In 28 reporting areas, by race or ethnicity, 80.1% of abortions obtained by Black women occurred at ≤9 weeks’ gestation, compared with 81.5% of non-Hispanic women in the other race category, 82.0% of White women, and 83.9% of Hispanic women. Conversely, 5.4% of abortions obtained by Hispanic women occurred after 13 weeks’ gestation, followed by 6.2% of White women, 6.3% of Black women, and 6.9% of non-Hispanic women in the other race category.

**TABLE 14:** Number of reported abortions, by known weeks of gestation, age group, and race or ethnicity — selected reporting areas, United States, 2020

Characteristic	Weeks of gestation							Total
	≤6	7–9	10–13	14–15	16–17	18–20	≥21	
	No. (%)	No. (%)	No. (%)	No. (%)	No. (%)	No. (%)	No. (%)	No. (%)
Age group (yrs)*^,†^								
<15	259 (25.6)	358 (35.4)	205 (20.3)	68 (6.7)	40 (4.0)	46 (4.5)	35 (3.5)	1,011 (100.0)
15–19	14,336 (37.9)	14,230 (37.6)	5,837 (15.4)	1,346 (3.6)	810 (2.1)	803 (2.1)	507 (1.3)	37,869 (100.0)
20–24	57,008 (43.7)	48,095 (36.9)	16,649 (12.8)	3,601 (2.8)	2,087 (1.6)	1,916 (1.5)	1,090 (0.8)	130,446 (100.0)
25–29	62,751 (46.3)	48,366 (35.7)	16,062 (11.8)	3,497 (2.6)	2,072 (1.5)	1,846 (1.4)	1,064 (0.8)	135,658 (100.0)
30–34	43,986 (47.5)	32,087 (34.6)	10,599 (11.4)	2,434 (2.6)	1,319 (1.4)	1,350 (1.5)	921 (1.0)	92,696 (100.0)
35–39	23,761 (47.5)	16,931 (33.9)	5,696 (11.4)	1,362 (2.7)	807 (1.6)	859 (1.7)	562 (1.1)	49,978 (100.0)
≥40	8,846 (50.7)	5,505 (31.6)	1,823 (10.5)	481 (2.8)	294 (1.7)	303 (1.7)	182 (1.0)	17,434 (100.0)
**Total**	**210,947 (45.4)**	**165,572 (35.6)**	**56,871 (12.2)**	**12,789 (2.7)**	**7,429 (1.6)**	**7,123 (1.5)**	**4,361 (0.9)**	**465,092 (100.0)**
Race or ethnicity*^,§^								
Non-Hispanic								
White	49,617 (47.3)	36,387 (34.7)	12,313 (11.7)	2,692 (2.6)	1,481 (1.4)	1,520 (1.4)	867 (0.8)	104,877 (100.0)
Black	55,749 (43.6)	46,628 (36.5)	17,308 (13.5)	3,608 (2.8)	1,972 (1.5)	1,809 (1.4)	714 (0.6)	127,788 (100.0)
Other	9,564 (47.4)	6,876 (34.1)	2,325 (11.5)	555 (2.8)	331 (1.6)	286 (1.4)	219 (1.1)	20,156 (100.0)
Hispanic	34,236 (51.6)	21,446 (32.3)	7,059 (10.6)	1,514 (2.3)	878 (1.3)	816 (1.2)	425 (0.6)	66,374 (100.0)
**Total**	**149,166 (46.7)**	**111,337 (34.9)**	**39,005 (12.2)**	**8,369 (2.6)**	**4,662 (1.5)**	**4,431 (1.4)**	**2,225 (0.7)**	**319,195 (100.0)**

* Percentages for the individual component categories might not add to 100% because of rounding.

^†^ Data from 41 reporting areas; excludes 11 reporting areas (California, Connecticut, the District of Columbia, Illinois, Maryland, Massachusetts, New Hampshire, New York State, Pennsylvania, Tennessee, and Wisconsin) that did not report, did not report weeks of gestation by age, or did not meet reporting standards.

^§^ Data from 28 reporting areas; excludes 24 reporting areas (California, Colorado, Connecticut, the District of Columbia, Hawaii, Illinois, Iowa, Louisiana, Maine, Maryland, Massachusetts, Nebraska, New Hampshire, New Jersey, New York City, New York State, North Dakota, Ohio, Oklahoma, Pennsylvania, Rhode Island, Tennessee, Washington, and Wisconsin) that did not report, did not report weeks of gestation by race or ethnicity, or did not meet reporting standards.

### Abortion Mortality

Using national PMSS data (*33*), CDC identified four abortion-related deaths for 2019, the most recent year for which data were reviewed for abortion-related deaths (Table 15). Investigation of these cases indicated all deaths were related to legal abortion.

**TABLE 15:** Number of deaths and case-fatality rates* for abortion-related deaths reported to CDC, by type of abortion — United States, 1973–2019^†^

Year	Type of abortion				CFR per 100,000 legal abortions
	Induced		Unknown**	Total	
	Legal^§^	Illegal^¶^			
**1973–1977**					**2.09**
1973	25	19	3	**47**	
1974	26	6	1	**33**	
1975	29	4	1	**34**	
1976	11	2	1	**14**	
1977	17	4	0	**21**	
**1978–1982**					**0.78**
1978	9	7	0	**16**	
1979	22	0	0	**22**	
1980	9	1	2	**12**	
1981	8	1	0	**9**	
1982	11	1	0	**12**	
**1983–1987**					**0.66**
1983	11	1	0	**12**	
1984	12	0	0	**12**	
1985	11	1	1	**13**	
1986	11	0	2	**13**	
1987	7	2	0	**9**	
**1988–1992**					**0.74**
1988	16	0	0	**16**	
1989	12	1	0	**13**	
1990	9	0	0	**9**	
1991	11	1	0	**12**	
1992	10	0	0	**10**	
**1993–1997**					**0.52**
1993	6	1	2	**9**	
1994	10	2	0	**12**	
1995	4	0	0	**4**	
1996	9	0	0	**9**	
1997	7	0	0	**7**	
**1998–2002**					**0.63**
1998	9	0	0	**9**	
1999	4	0	0	**4**	
2000	11	0	0	**11**	
2001	7	1	0	**8**	
2002	10	0	0	**10**	
**2003–2007**					**0.60**
2003	10	0	0	**10**	
2004	7	1	0	**8**	
2005	7	0	0	**7**	
2006	7	0	0	**7**	
2007	6	0	0	**6**	
**2008–2012**					**0.65**
2008	12	0	0	**12**	
2009	8	0	0	**8**	
2010	10	0	0	**10**	
2011	2	0	0	**2**	
2012	4	0	0	**4**	
**2013–2019**					**0.43**
2013	4	0	0	**4**	
2014	6	0	0	**6**	
2015	2	0	1	**3**	
2016	6	1	1	**8**	
2017	3	0	0	**3**	
2018	2	0	0	**2**	
2019	4	0	0	**4**	

**Abbreviation:** CFR = case-fatality rate.

* Number of legal induced abortion-related deaths per 100,000 reported legal induced abortions. Because a substantial number of legal induced abortions occurred outside reporting areas that provided data to CDC, national CFRs (i.e., number of legal induced abortion-related deaths per 100,000 reported legal induced abortions in the United States) were calculated with denominator data from the Guttmacher Institute’s national survey of abortion-providing facilities. Case-fatality rates were computed for consecutive 5-year periods during 1973–2012 and then for a consecutive 7-year period during 2013–2019 because rates based on <20 cases might be unstable.

^†^ Certain numbers might differ from those in reports published previously because additional information has been supplied to CDC subsequent to publication.

^§^ An abortion is defined as legal if it was performed by a licensed clinician within the limits of state law.

^¶^ An abortion is defined as illegal if it was performed by any person other than a licensed clinician.

** Unknown whether abortion was induced or spontaneous.

The annual number of deaths related to legal induced abortion has fluctuated from year to year since 1973 (Table 15). The national case-fatality rate for legal induced abortion for 2013–2019 was 0.43 deaths related to legal induced abortions per 100,000 reported legal abortions. This case-fatality rate was lower than the rates for the previous 5-year periods.

## Discussion

For 2020, a total of 620,327 abortions were reported to CDC by 49 areas. Among the 48 continuously reporting areas, for 2020, the abortion rate was 11.2 abortions per 1,000 women aged 15–44 years, and the abortion ratio was 198 abortions per 1,000 live births. From 2019 to 2020, the number of abortions decreased 2%, the abortion rate decreased 2%, and the abortion ratio increased 2%. Although the rate of reported abortions declined overall from 2011 to 2020, after reaching a historic low in 2017, the overall abortion rate increased between 2018 and 2019, before declining again in 2020.

Using data from their national survey of abortion-providing facilities, the Guttmacher Institute estimated that approximately 21% of all pregnancies in the United States ended in induced abortion in 2020 (*34*). Multiple factors influence the incidence of abortion, including access to health care services and contraception (*36*–*38*); the availability of abortion providers and clinics (*6*,*39*,*40*); state regulations, such as mandatory waiting periods (*41*–*43*), parental involvement laws (*44*,*45*), and legal restrictions on abortion providers and clinics (*46*–*52*); and changes in the economy and the resulting impact on family planning decisions and contraceptive use (*53*).

Abortion measures in 2020 might have been affected by the COVID-19 pandemic. Factors include temporary changes that defined abortion as a nonessential service at the hospital, local, or jurisdiction level (*54*,*55*), clinic closures, and changes in practice (e.g., shift from surgical abortions to medical abortions, implementation, and uptake of telehealth) (*56*–*60*). In addition, there might have been changes in pregnancy rates because of reduced sexual activity (*61*,*62*).

Abortion measures also differ by demographic characteristics. Among areas that reported data continuously by age during 2011–2020, women in their 20s accounted for the highest percentages of abortions and had the highest abortion rates, whereas adolescents aged <15 years accounted for the lowest percentage of abortions and had the lowest abortion rate, and adolescents aged <15 years and 15–19 years had the highest abortion ratios. During 2011–2020, women aged ≥40 years accounted for a relatively small percentage of reported abortions (≤3.8%). However, the abortion ratio among women aged ≥40 years continues to be higher than among women aged 25–39 years.

The percentage change in adolescent abortions described in this report are important for monitoring changes in adolescent pregnancies in the United States. From 2011 to 2020, national birth data indicate that the birth rate for adolescents aged 15–19 years decreased 51% (*31*), and the data in this report indicate that the abortion rate for the same age group decreased 48%. These findings highlight that decreases in adolescent births in the United States have been accompanied by large decreases in adolescent abortions (*31*).

As in previous years, abortion rates and ratios differ across racial or ethnic groups. For example, in 2020, compared with White women, abortion rates and ratios were 3.9 and 3.6 times higher among Black women and 1.8 and 1.5 times higher among Hispanic women. Similar differences by race or ethnicity have been demonstrated in other U.S.-based studies (*2*,*8*–*11*,*63*). The factors leading to higher abortion rates among certain racial or ethnic minority groups are complex. In addition to disparities in rates of unintended pregnancies, structural factors, including unequal access to quality family planning services (*64*,*65*), economic inequities, and mistrust of the medical system (*66*), can contribute to observed differences.

In 2020, approximately four out of five abortions occurred early in gestation (≤9 weeks), when the risks for complications are lowest (*67*–*70*). Over the past 10 years, this percentage increased from 74.3% in 2011 to 79.1% in 2020. Moreover, among areas that reported abortions at ≤13 weeks’ gestation by individual week, the distribution of abortions by gestational age continued to shift toward earlier weeks of gestation, with the percentage of early abortions performed at ≤6 weeks’ gestation increasing from 34.2% in 2011 to 39.9% in 2020. Previous research indicates that the distribution of abortions by gestational age differs by various sociodemographic characteristics (*71*–*73*). In this report, the percentage of adolescents aged ≤19 years who obtained abortions at >13 weeks’ gestation was higher than the percentage among women aged ≥20 years. The gestational age when abortions are performed can be influenced by multiple factors, including jurisdiction abortion restrictions, accurate estimation of gestational age, income level, age, and presence of pregnancy-related health conditions (*41*,*63*,*70*,*72*–*77*).

Changes in clinical practices have facilitated the trend of obtaining abortions earlier in pregnancy. Research conducted in the United States during the 1970s indicated that surgical abortion procedures performed at ≤6 weeks’ gestation, compared with 7–12 weeks’ gestation, were less likely to result in successful termination of the pregnancy (*78*). However, subsequent advances in technology (e.g., improved transvaginal ultrasonography and sensitivity of pregnancy tests) have allowed very early surgical abortions to be performed with completion rates exceeding 97% (*79*–*82*). Likewise, the development of early medical abortion regimens has allowed for abortions to be performed early in gestation, with completion rates for regimens that combine mifepristone and misoprostol reaching 96%–98% (*82*–*85*).

Trends for early medical abortions are reported to monitor any changes in clinical practice that might have occurred with the accumulation of evidence on the safety and effectiveness of medical abortion past 63 days of gestation (8 completed weeks’ gestation) (*86*), changes in professional practice guidelines published in 2013 and 2014 (*87*,*88*), and the 2016 FDA extension of the gestational age limit for the use of mifepristone for early medical abortion from 63 days to 70 days (9 completed weeks’ gestation) (*89*). Among abortions occurring at ≤9 weeks’ gestation in 2020, 63.9% of abortions were reported as early medical abortions. In 2020, the most common method among abortions reported overall was early medical abortion at ≤9 weeks’ gestation (51.0%). Among areas that reported by method type and included medical abortion on their reporting form, the percentage of all abortions performed by early medical abortion increased 154% from 2011 to 2020 and increased 22% from 2019 to 2020.

Because the annual number of deaths related to legal induced abortion is small and statistically unstable, case-fatality rates were calculated for consecutive 5-year periods during 1973–2012 and then for a consecutive 7-year period during 2013–2019. The national case-fatality rate for legal induced abortion was 0.43 per 100,000 abortions. Since the late 1970s, all rates for the preceding 5-year periods have been fewer than 1 death per 100,000 abortions, demonstrating the low risk for death associated with legal induced abortion.

## Limitations

The findings in this report are subject to at least four limitations. First, because reporting to CDC is voluntary and reporting requirements vary by the individual reporting areas (*13*,*14*), CDC is unable to report the total number of abortions performed in the United States. Of the 52 areas from which CDC requested data for 2020, California, Maryland, and New Hampshire did not submit abortion data. In 2020, the most recent year for which data are available through the Guttmacher Institute’s national survey of abortion-providing facilities, abortions performed in these states accounted for approximately 20% of all abortions in the United States (*34*). CDC receives aggregated data from the central health agencies of reporting areas, which might result in different estimates than reported by the Guttmacher Institute. New Jersey did not have abortion reporting requirements to a centralized health agency during the period covered in this report (*13*), which potentially affects the representativeness of data provided to CDC. Certain reporting areas (the District of Columbia and Wyoming) have recently implemented new legislation that could improve reporting of abortion data. Nonetheless, even in reporting areas that legally require clinicians to submit a report for every abortion they perform, enforcement of this requirement varies.

Second, many states use abortion reporting forms that differ from the technical guidance that CDC developed in collaboration with NAPHSIS. Consequently, certain reporting areas do not collect all variables requested by CDC (e.g., age and race or ethnicity) or do not report the data in a manner consistent with this guidance (e.g., gestational age). Missing demographic information can reduce the extent to which the statistics in this report represent women who have had abortions. Only 30 reporting areas reported race or ethnicity data to CDC that met CDC’s reporting standards. Certain areas that either do not report to CDC (e.g., California) or do not report race or ethnicity data (e.g., Illinois) have sufficiently large populations of racial or ethnic minority groups that the absence of data from these areas likely reduces the representativeness of CDC data for these variables. In addition, because of the variability in data collection for race or ethnicity among reporting areas, data for specific racial or ethnic groups beyond White, Black, and Hispanic are not requested or reported. In addition, certain areas collect gestational age data that are based on estimated date of conception or probable postfertilization age, which are not consistent with medical conventions for gestational age reporting. Without medical guidance on how to report these data, the validity and reliability of gestational age for these reporting areas is uncertain.

Third, abortion data are compiled and reported to CDC by the central health agency of the reporting area in which the abortion was performed rather than the reporting area in which the person lived. Thus, the available population (*20*–*29*) and birth data (*30*,*31*), which are organized by the states/jurisdictions in which women live, might differ from the population of women who undergo abortions in a given reporting area. This likely results in an overestimation of abortions for reporting areas in which a higher percentage of abortions are obtained by out-of-area residents and an underestimation of abortions for reporting areas where residents more frequently obtain abortions out of area. Limited abortion services, stringent regulatory requirements for obtaining an abortion, or geographic proximity to services in another state might influence where women obtain abortion services (*90*,*91*).

Finally, CDC reporting of sociodemographic characteristics of women obtaining abortions is limited to data collected on jurisdiction reporting forms. Therefore, the examination of additional demographic variables (e.g., income and education) is not possible.

## Public Health Implications

Ongoing surveillance of legal induced abortion is important for several reasons. First, abortion surveillance can be used to help evaluate programs aimed at promoting equitable access to patient-centered contraceptive care in the United States to reduce unintended pregnancies. Up to 42% of unintended pregnancies in the United States end in abortion (*92*), and use of effective contraception is a strategy to reduce unintended pregnancy (*93*). Efforts to improve contraceptive access have been associated with declines in the rate of abortions (*36*,*38*). Reported barriers to accessing contraception include cost, inadequate provider reimbursement and training, insufficient patient-centered counseling, lack of youth-friendly services, and low client awareness of available contraceptive methods (*36*–*38*,*94*–*100*). Reducing these barriers might help ensure equitable access to patient-centered contraceptive care and promote equitable reproductive health in the United States (*101*).

Second, routine abortion surveillance can be used to assess changes in clinical practice patterns over time. Information in this report on the number of abortions performed through different methods (e.g., medical or surgical) and at different gestational ages provides the denominator data that are necessary for analyses of the relative safety of abortion practices (*102*,*103*). Finally, information on the number of pregnancies ending in abortion is used in conjunction with data on births and fetal losses to estimate the number of pregnancies in the United States and determine rates for various outcomes of public health importance (*12*).
